# An RGD motif on SARS-CoV-2 Spike induces TGF-β signaling and downregulates interferon

**DOI:** 10.1128/jvi.00435-25

**Published:** 2025-09-04

**Authors:** Nicholas P. Gracie, Anupriya Aggarwal, Rachel Luo, Mitchell Spicer, Sobia Idrees, Caroline L. Ashley, Sibel Alca, Timothy Ison, Megan C. Steain, Karishma Patel, Rezwan Siddiquee, Jason K. K. Low, Joel P. Mackay, Christopher E. Denes, G. Gregory Neely, Alen Faiz, Stuart G. Turville, Timothy P. Newsome

**Affiliations:** 1 School of Life and Environmental Sciences, The University of Sydney, Camperdown, New South Wales, Australia; 2 Kirby Institute, University of New South Wales 2786 , Kensington, New South Wales, Australia; 3 Respiratory Bioinformatics and Molecular Biology (RBMB), School of Life Sciences, University of Technology Sydney 1994, Sydney, New South Wales, Australia; 4 Centre for Inflammation, Faculty of Science, School of Life Sciences, Centenary Institute and University of Technology Sydney, Sydney, New South Wales, Australia; 5 Infection, Immunity and Inflammation, School of Medical Sciences, Faculty of Medicine and Health, The University of Sydney, Camperdown, New South Wales, Australia; 6 Sydney Institute of Infectious Diseases (Sydney ID), The University of Sydney, Camperdown, New South Wales, Australia; 7 The Dr John and Anne Chong Lab for Functional Genomics, School of Life & Environmental Sciences, Charles Perkins Center, The University of Sydney 4334, Sydney, New South Wales, Australia; The Ohio State University, Columbus, Ohio, USA

**Keywords:** SARS-CoV-2, TGF-β, coronavirus, COVID-19, RGD (Arg-Gly-Asp), integrins, PAI-1, IFN-β

## Abstract

The severe acute respiratory syndrome coronavirus 2 (SARS-CoV-2) spike (S) protein mediates canonical cell entry via ACE2 and has also been implicated as an activator of a diverse range of signaling pathways. Here, we present evidence that the RGD (Arg-Gly-Asp) motif within the receptor-binding domain (RBD) of the S1 fragment of the S protein induces TGF-β cytokine expression. RGD peptides are well characterized as ligands for a subset of integrin complexes primarily containing α5 and αV subunits. In this study, we investigate the molecular basis of TGF-β pathway activation by S protein, delivered to cells as recombinant protein, in pseudotyped virus or in virally infected cells. Activation of TGF-β signaling by the S protein requires ACE2 and leads to SMAD3-dependent expression of the pro-fibrotic marker PAI-1. Utilizing pseudotyped viruses, expression of the S protein with a mutated RGD motif abolished TGF-β signaling, as did the RGD antagonist ATN-161, implicating integrin complexes in mediating this response. We show that the S protein RGD motif suppresses IFN-β expression via TGF-β, leading to a disruption in cellular antiviral defenses, consistent with TGF-β’s role in immunosuppression. These findings further support the multifunctionality of S protein and provide mechanistic insights into its activity as a virulence factor during infection.

**IMPORTANCE:**

Severe acute respiratory syndrome coronavirus 2 (SARS-CoV-2) presents an ongoing public health challenge as a cause of acute illness and post-acute sequelae of COVID-19 (PASC, or long COVID). Our study identifies the RGD integrin-binding motif in the spike (S) protein as central to the cellular response to SARS-CoV-2, leading to the expression of the pleiotropic cytokine TGF-β and disabling of antiviral immunity. This work further supports the S protein-to-integrin complex signaling axis as a potential therapeutic target. The RGD motif might also be a valid target for treating PASC given the increasing body of evidence implicating the presence of persistent S protein in the etiology of this disease.

## INTRODUCTION

The *Coronaviridae* family comprises important human pathogens including endemic human respiratory viruses (OC43, HKU1, 229E, NL63) and highly virulent zoonotic pathogens SARS-CoV and MERS-CoV. Novel severe acute respiratory syndrome (*SARS)-like coronavirus-2* (SARS-CoV-2), the viral cause of the Coronavirus disease 2019 (COVID-19), belongs to the *Sarbecovirus* subgenus within this family (1). All coronaviruses share a common morphology with four structural proteins, including the spike (S) envelope glycoprotein (2). The S protein, present in trimers on the viral surface, is essential for mediating viral entry into host cells through two key events: cleavage and receptor binding. Cleavage of S protein by cellular furin-like proteases releases subunits S1 and S2. S1 contains the N-terminal domain (NTD) and the receptor-binding domain (RBD), which binds directly to the host cell receptor ACE2, the first step in canonical entry (2, 3). S2 contains the fusion peptide, which is activated after the second cleavage of S2 by the cellular serine protease TMPRSS2, and this enables delivery of the genome to the cytoplasm (2–6). S protein is the core component of all SARS-CoV-2 vaccines, and targeting this protein has proven highly effective in generating effective neutralizing antibodies, reducing transmission, infection, and hospitalization (7–9).

The S protein includes an RGD motif (Arg-Gly-Asp; residues 403–405) located in the RBD of S1, a motif present in a variety of extracellular matrix (ECM) proteins that interact with heterodimeric integrin complexes comprising two α and two β subunits. The RGD motif is a ligand for a subset of integrin complexes, principally those containing αV and α5 subunits (10–12). A functionally related KGD (Lys-Gly-Asp; residues 390–392) motif is present on the S protein of SARS-CoV, the cause of the 2003 epidemic, which is also a Sarbecovirus that infects cells via ACE2-mediated entry (13, 14). The KGD motif interacts with an extended albeit broadly similar set of integrin complexes as the RGD motif. The negatively charged aspartic acid residue in RGD and KGD motifs is critical for the interaction with the positive metal ion-dependent adhesion site (MIDAS) on the top face of the β integrin, while the arginine binds strongly with the α subunit, both forming a binding pocket in the integrin complex (15–17). For example, fibronectin engages α5β1 and αvβ3 integrins through this interaction between the binding pocket and the RGD motif, leading to activation and conformational changes in the integrin complex (16, 18). Some integrins can be primed prior to this binding to an extended open state (EOS) usually through the presence of cations such as manganese (Mn^2+^) (19, 20). Following ligand binding, integrin complexes can drive bidirectional signaling, allowing for engagement with multiple signaling pathways and eliciting a range of cellular effects including attachment, inflammation, and cell-to-cell interactions (19, 20).

Integrins have been implicated in SARS-CoV-2 infection, but the specific roles and mechanisms of these complexes remain varied and disputed. This is evidently seen in the role of integrins in SARS-CoV-2 entry (14, 21, 22). Forced EOS integrins via Mn^2+^ treatment have been demonstrated to aid in SARS-CoV-2 cell entry and additionally assist in intracellular trafficking (23, 24). Additionally, an enrichment of αvβ1 and αvβ3 integrin complexes is observed during SARS-CoV-2 infection (21, 25). During infection, these integrin complexes have been shown to work in tandem with ACE2 to mediate viral entry, leading to additional cell line-specific effects such as coagulopathy (21, 26, 27). Many of these studies link the RGD motif in S protein to these integrin functions. The specificity of these interactions has been shown experimentally by RGD loss-of-function mutations, pretreatment of cells with RGD peptides, anti-integrin neutralizing antibodies, or via the use of an RGD antagonist such as ATN-161 (13, 21–23, 25). Specifically, the use of ATN-161 rescues transgenic ACE2-expressing mice from a lethal dose of SARS-CoV-2 (28). *In vitro* studies demonstrate to a similar effect that utilizing ATN-161 to prevent RGD-binding to integrins can rescue cells from S protein-induced pathology (29–31).

In addition to viral entry, the S protein is implicated in a range of signaling events during SARS-CoV-2 infection (32). One example is transforming growth factor β (TGF-β), whose levels are elevated following treatment of cells with recombinant S protein (33). TGF-β is a pleiotropic cytokine associated with homeostasis, immune regulation, and cellular repair (34). Importantly, latent TGF-β is induced in the presence of αV and α5 integrins, to enact canonical TGF-β signaling (35–37). In COVID-19, elevated serum and blood plasma TGF-β levels correlate with disease severity, as indicated by increased levels of downstream pro-fibrotic marker PAI-1 (38–42). This upregulated TGF-β signaling has been shown to diminish the function of NK cells, weakening antiviral activity and impairing B-cell function (40, 43). Biering et al. demonstrated that S protein induces TGF-β expression, resulting in endothelial vascular dysregulation and barrier dysfunction (33). Endothelial cell barrier dysfunction induced by S protein could be rescued by treatment with the TGF-β inhibitor SB-431542 or ATN-161 (33). These observations suggest that the S protein RGD motif may play a critical role in driving TGF-β-induced pathology via integrin complexes.

The TGF-β pathway has previously been suggested as a potential drug target to mitigate some of the adverse consequences of infection, and a growing body of molecular studies is providing more credibility for this approach. Since TGF-β is a potent immune suppressor, localized signaling by Spike may stimulate significant alterations to the immune response (44, 45). In addition to reducing type I IFNs and overexpression of pro-inflammatory pathways, TGF-β invokes the differentiation of naive T-cells to regulatory T-cells, leading to a reduction in dendritic cells and M1 proinflammatory macrophages (46–51). This potential to disentangle the antigenic and signaling functions of the RGD motif may represent a mechanism to optimize antiviral design and reduce the risk of reinfection.

In our study, we investigate the molecular basis of TGF-β pathway activation by S protein. We show that S protein induces TGF-β expression, activates TGF-β pathway reporter assays, promotes the expression of the fibrotic marker PAI-1, and is able to suppress the induction of the antiviral cytokine IFN-β. The TGF-β transcriptional response induced by S protein was dependent on ACE2 and is inhibited by the RGD antagonist ATN-161 and the TGF-β pathway inhibitor SB-431542. Mutating the RGD motif in S protein ablates induction of TGF-β and suppression of IFN-β. Our results build on previous work, providing a mechanistic understanding of S protein induction of TGF-β by the RGD motif. We propose that TGF-β signaling during SARS-CoV-2 infection renders cells more susceptible to infection and may contribute to COVID-19 pathologies.

## RESULTS

### SARS-CoV-2 activates TGF-β signaling leading to PAI-1 expression

**Fig 1:**
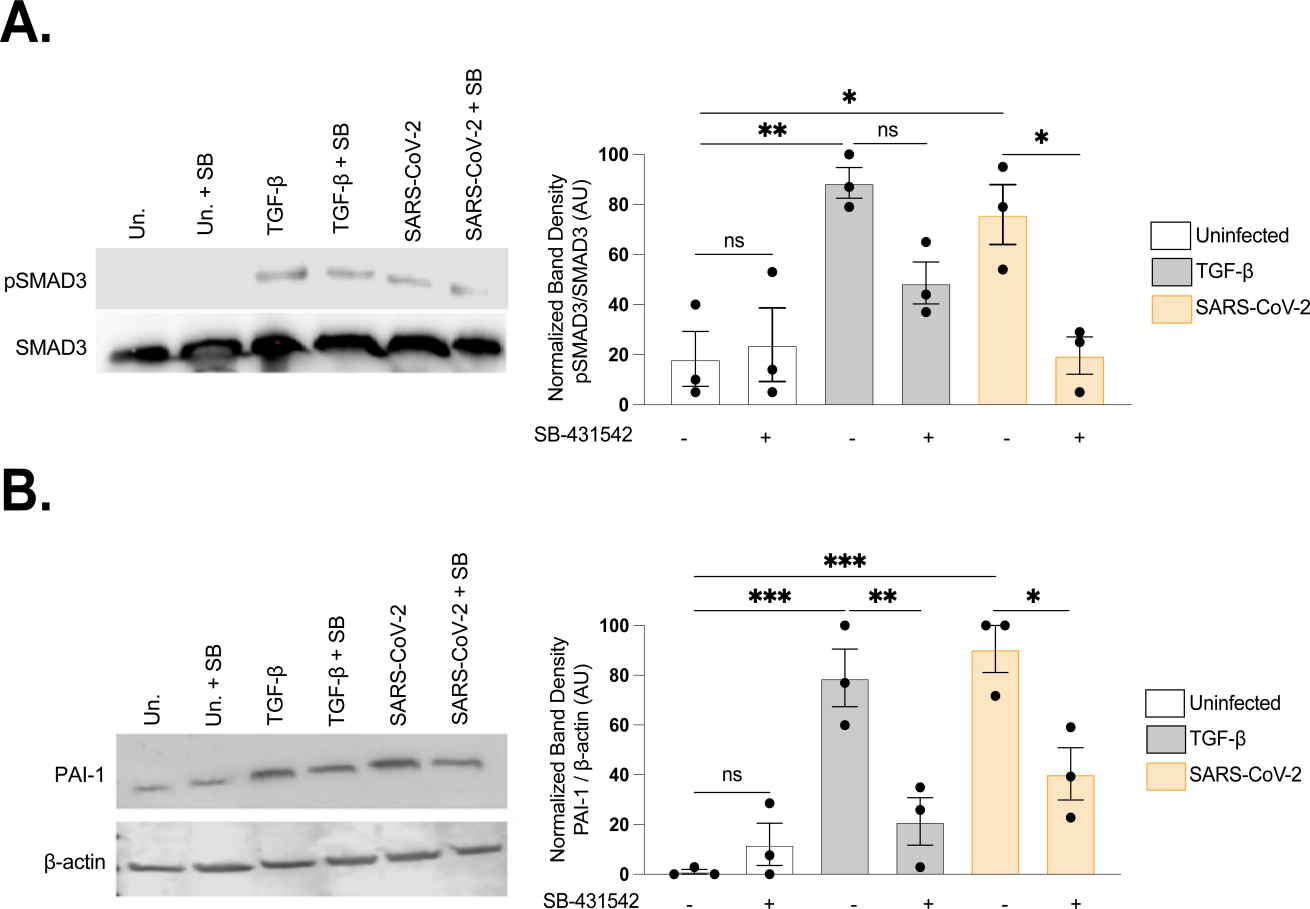
SARS-CoV-2 infection induces PAI-1 expression and SMAD3/4 complex activity, both reduced by TβR1 inhibition. Expression of phosphorylated SMAD3 and SMAD3 (**A**) PAI-1, β-actin (**B**), was measured via immunoblot in Calu-3 cells following 72 h infection with SARS-CoV-2 (Ancestral Strain A.2.2.; MOI = 0.1), treatment with TGF-β (2 ng/mL) or from untreated cells. From each condition, a parallel set of cells were treated 24 h prior to infection and during infection with TβR1 inhibitor SB-431542 (10 µM). Normalized band intensity for each immunoblot was calculated using FIJI (ImageJ2) ([**A**] *n* = 3, **P < 0.01 [**B**] n =3, ****P < 0.0001 both by one-way ANOVA with Tukey’s multiple comparison test).

Canonical TGF-β cellular signaling is driven by SMAD transcription factors. R-SMADs (SMAD2 and SMAD3) are phosphorylated at their C-termini by the TGF-β-receptor complex (TβRI and TβRII), leading to the formation of trimeric complexes with SMAD4 (Co-SMAD) that enter the nucleus and modulate gene expression (44, 45). To examine TGF-β signaling in SARS-CoV-2 replication, we assayed infected human lung adenocarcinoma/epithelial cells (Calu-3). Calu-3 cells were infected with SARS-CoV-2 (Ancestral Strain) at an MOI of 0.1 and lysed at 72 hpi. SARS-CoV-2 infection led to an increase in SMAD3 phosphorylation, which was inhibited by the TβRI inhibitor SB-431542 (Fig. 1A). Previous work has shown elevated PAI-1 transcripts (SERPINE1) in response to SARS-CoV-2 (33, 39, 41, 46). Consistent with activation of TGF-β signaling by SARS-CoV-2, PAI-1 was induced during infection, and this induction was inhibited by SB-431542 (Fig. 1B).

### SARS-CoV-2 S protein is sufficient to induce TGF-β expression and signaling.

**Fig 2:**
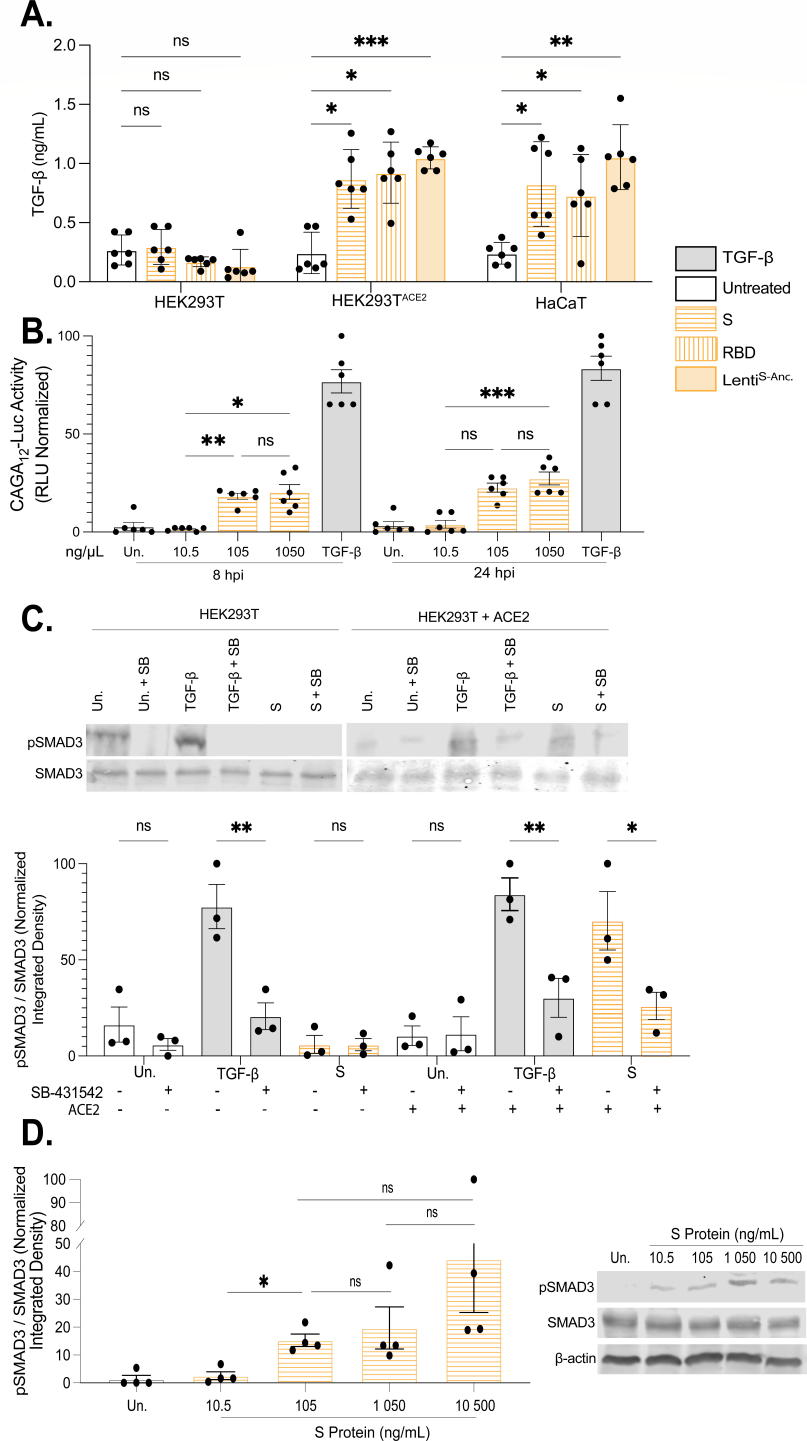
S protein induces TGF-β expression and activates SMAD3 activity. (**A**) TGF-β expression was detected by ELISA in cells treated with S protein (105 ng/mL) or RBD (200 ng/mL, 24h) or infected with S pseudotyped lentivirus (Lenti^S-Anc^, 24h) (n = 6, ***P < 0.01 by two-way ANOVA with Tukey’s multiple comparison test). (**B**) Activity of the transfected CAGA_12_-Luc (SMAD3/4) reporter elements in HEK293T^+ACE2^ cells was assayed following treatment with the indicated concentrations of S protein at 8 h and 24 h. (*n* = 4, ****P < 0.0001 by two-way ANOVA with Tukey’s multiple comparison test). (**C**) Phosphorylation of SMAD3 at the SXSS motif in response to S protein was determined by immunoblot in HEK293T^+ACE2^ and HEK293T^WT^ cells. Subsequently, half of these treated cells were supplemented with ALK kinase inhibitor SB-431542 (10 µM). Normalized band intensity for each immunoblot was calculated using FIJI (ImageJ2). (*n* = 3, ****P < 0.0001 by two-way ANOVA with Tukey’s multiple comparison test). (**D**) Phosphorylation of SMAD3 at the SSXS motif in response to different concentrations of S protein (10.5 ng/mL, 105 ng/mL, 1050 ng/mL, and 10500 ng/mL) was assessed via immunoblot in HaCaT Cells at 24 h. Normalized band intensity, to control SMAD3 for each immunoblot, was calculated using FIJI (ImageJ2) (n = 4, *p<0.05 by one-way ANOVA with Tukey’s multiple comparison test).

We next sought to determine the role for S protein in the activation of TGF-β signaling and PAI-1 induction as S protein is sufficient to trigger TGF-β induction (33, 52). We treated cells with recombinantly expressed SARS-CoV-2 S protein ectodomain (S protein; residues 1–1,208 derived from Wuhan strain D614G, HexaPro variant) or the SARS-CoV-2 S protein receptor-binding domain (RBD; residues 319–541) and examined TGF-β expression by ELISA (53, 54). HaCaT cells were selected as they represent a well-established model for assaying TGF-β signaling, and these cells natively express ACE2 (55–58). ACE2-expressing HEK293T (HEK293T^+ACE2^) cells and non-ACE2-expressing HEK293T (HEK293T^WT^) cells were included in these experiments to determine the requirement for ACE2 (59). TGF-β was induced following S protein or RBD treatment and was dependent on ACE2 expression, indicating that the activity of S protein maps to the RBD (Fig. 2A).

In support of this, SARS-CoV-2 S protein delivered to cells by pseudotyped replication-defective lentivirus (Lenti^S-Anc^) led to TGF-β expression (Fig. 2A). S protein led to activation of the SMAD3 (CAGA_12_) luciferase reporter assay, indicating an optimal concentration of 105 ng/mL consistent with activation of TGF-β signaling (Fig. 2B, *P* < 0.01). These results seemed somewhat surprising since the concentration of S protein required for pathway activity was 10-fold lower than what had previously been shown to activate TGF-β (33). Additionally, S protein was sufficient to induce SMAD3 phosphorylation, which was ACE2-dependent and inhibited by SB-431542 (Fig. 2C). The same novel concentration of S protein (105 ng/mL) also provided a significant increase in SMAD3 phosphorylation, where increasing the dosage increased this activity (Fig. 2D, *P* < 0.05). SMAD3 phosphorylation also occurred during Lenti^S-Anc^ transduction. This activity induced by Lenti^S-Anc^ peaked at 24 hpi (Fig. S1A and B).

**Fig 3:**
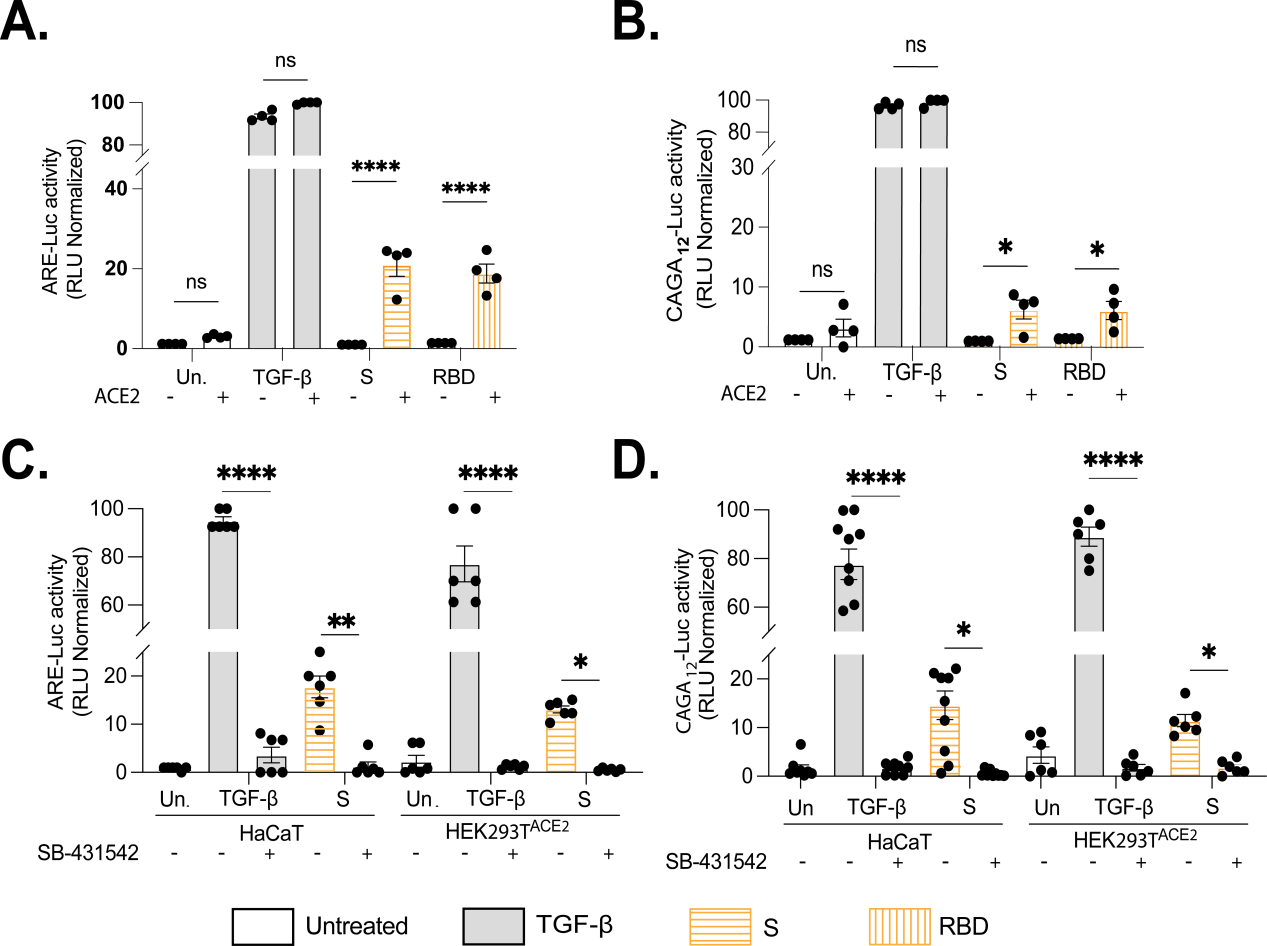
S protein and RBD activate SMAD2/SMAD3 in an ACE2- and TβR1-dependent manner (**A**) SMAD2/4 (ARE-Luc) and (**B**) SMAD3/4 (CAGA_12_-Luc) reporters were assayed at 24 h in S protein and RBD-treated cells that express (HEK293T^+ACE2^) or do not express (HEK293T) ACE2 ([**A**] n = 4, ****P < 0.0001 [**B**] n = 4, ***P < 0.001 by one-way ANOVA with Tukey’s multiple comparison test). Activity of the transfected (**C**) SMAD2/4 (ARE-Luc) and (**D**) SMAD3/4 (CAGA_12_-Luc) reporter elements in HaCaT^WT^ and HEK293T^+ACE2^ was measured following treatment with either TGF-β (2 µg/mL), S (105 ng/mL), or RBD Protein (200 ng/mL) or left untreated for 24 h. Subsequently, half of these treated cells were supplemented with ALK kinase inhibitor SB-431542 (10 µM). Data were normalized relative to positive (TGF-β) and negative (non-transfected, Renilla) controls. ([**C**] n = 6, ****P < 0.0001, [**D**] n = 6, ****P < 0.0001 by two-way ANOVA with Tukey’s multiple comparison test).

Having established that S protein promotes R-SMAD3 phosphorylation and activity, we expanded our analysis to include additional SMAD luciferase reporters. Both full-length S protein and the RBD stimulated SMAD2/4 (ARE-Luc), SMAD3 (SBR_6_-Luc), and SMAD3/4 (CAGA_12_-Luc) reporter assays and were dependent on ACE2 (Fig. 3A and B; Fig. S2). While S protein modestly increased SBR_6_-Luc activity in ACE2-expressing cells, the difference compared to cells lacking ACE2 was not statistically significant (Fig. S2). Activation of ARE-Luc and CAGA_12_-Luc reporter assays requires the activity of TβRI, which was inhibited by SB-431542 (Fig. 3C and D).

**Fig 4:**
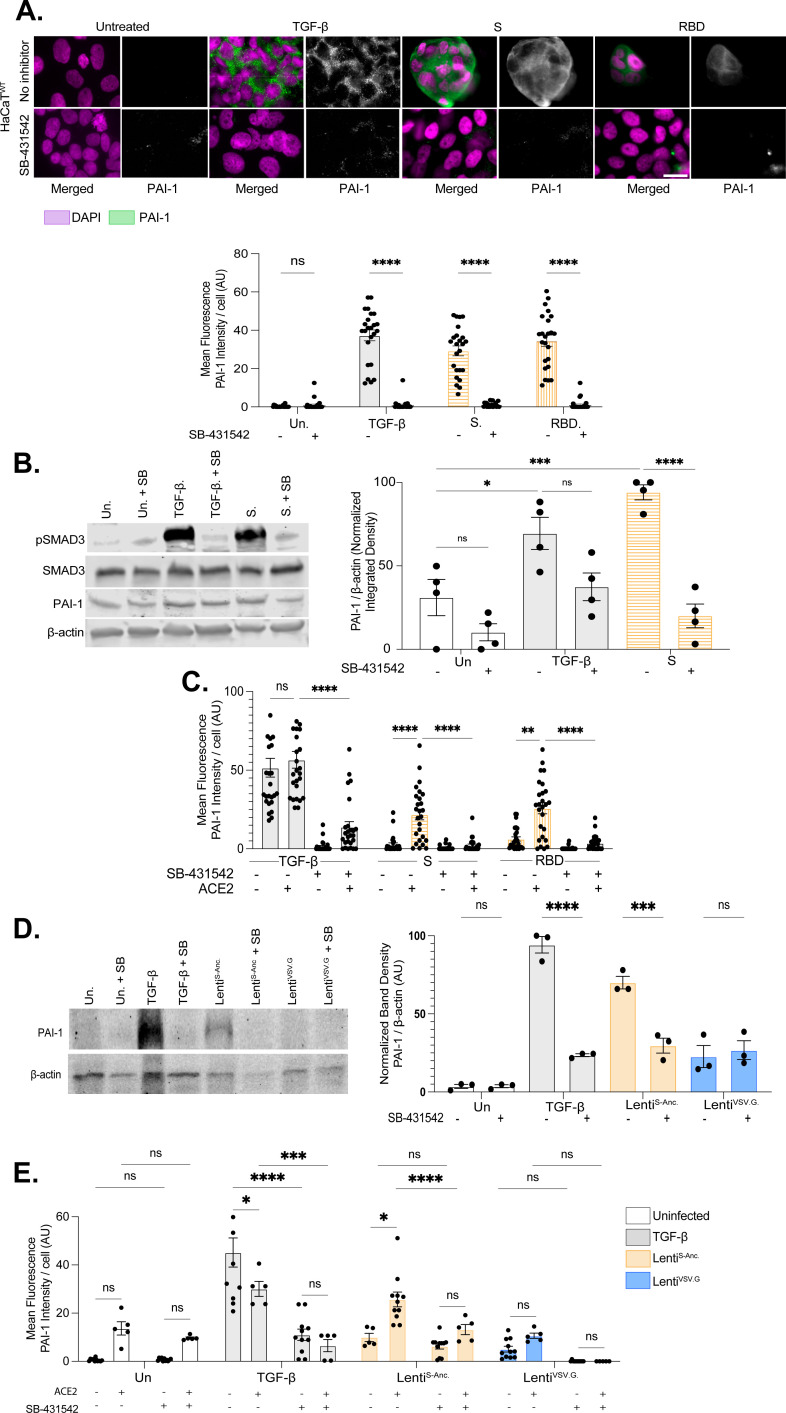
S protein induction of PAI-1 expression. Immunofluorescence micrographs of (**A**) HaCaT^WT^, (**C**) HEK293T^WT,^ or HEK293T^+ACE2^ cells (micrographs are found in [Fig. S2]) treated with TGF-β (2 ng/mL), S protein (105 ng/mL), or RBD (200 ng/mL) or left untreated for 24 h. Additionally, half of the treated cells were supplemented with SB-431542 (10 µM) 1 h prior to treatment. SMAD3-dependent protein PAI-1 (green) and nucleic acid (DAPI, magenta) were visualized. Scale bar = 50 µm. Mean PAI-1 fluorescence per nuclei was quantified. ([**A**] n = 24 cells [**C**] n = 26, both representative of three independent experiments, ****P < 0.0001 by two-way ANOVA with Tukey’s multiple comparisons test). (**B, D**) Immunoblots showing PAI-1 expression in HaCaT^WT^ cells treated with TGF-β (2 ng/mL), S protein (105 ng/mL), (**D**) transduced with lenti^S-Anc^ or lenti^VSV.G^, or left untreated. Additionally, half of the treated cells were supplemented with SB-431542 (10 µM) 1 h prior to treatment. β-actin was used as a loading control. Band intensities were quantified using FIJI. ([**B**] n = 5, ****P < 0.0001; [**D**] n=3, ***P < 0.001, ANOVA with Tukey’s multiple comparisons test). (**E**) Quantification of PAI-1 fluorescence in HEK293T^WT^ and HEK293T^+ACE2^ cells treated in (**A, C**) or transduced with lenti^S-Anc.^ or lenti^VSV.G^ for 24 h. Data representative of six technical replicates across three biological replicates. (n = 3; ***P < 0.001, two-way ANOVA with Tukey’s multiple comparisons test).

TGF-β is sufficient to induce PAI-1 expression assayed by immunofluorescence and immunoblot assay in HaCaT^WT^ cells, and this activity is replicated by S protein and RBD (Fig. 4A and B). PAI-1 induction by S protein and RBD was ACE2-dependent and inhibited by SB-431542, indicating the role of the TGF-β pathway (Fig. 4C). We observed that ACE2-expressing cells treated with Lenti^S-Anc^, but not the VSV.G control (Lenti^VSV.G^), significantly induced PAI-1 expression. Consistent with our previous findings, induction of PAI-1 was inhibited by the TβR1 inhibitor SB-431542 (Fig. 4D and E).

We next addressed whether the activity of recombinantly expressed S protein was replicated in our S pseudotyped virus model. We observed that ACE2-expressing cells treated with Lenti^S-Anc^ induced PAI-1 expression (Fig. 4D and E). Consistent with our previous findings, induction of PAI-1 was inhibited by the TβR1 inhibitor SB-431542. Additionally, the control VSV.G pseudotyped lentivirus (Lenti^VSV.G^) was unable to induce PAI-1 potently (Fig. 4D and E).

**Fig 5:**
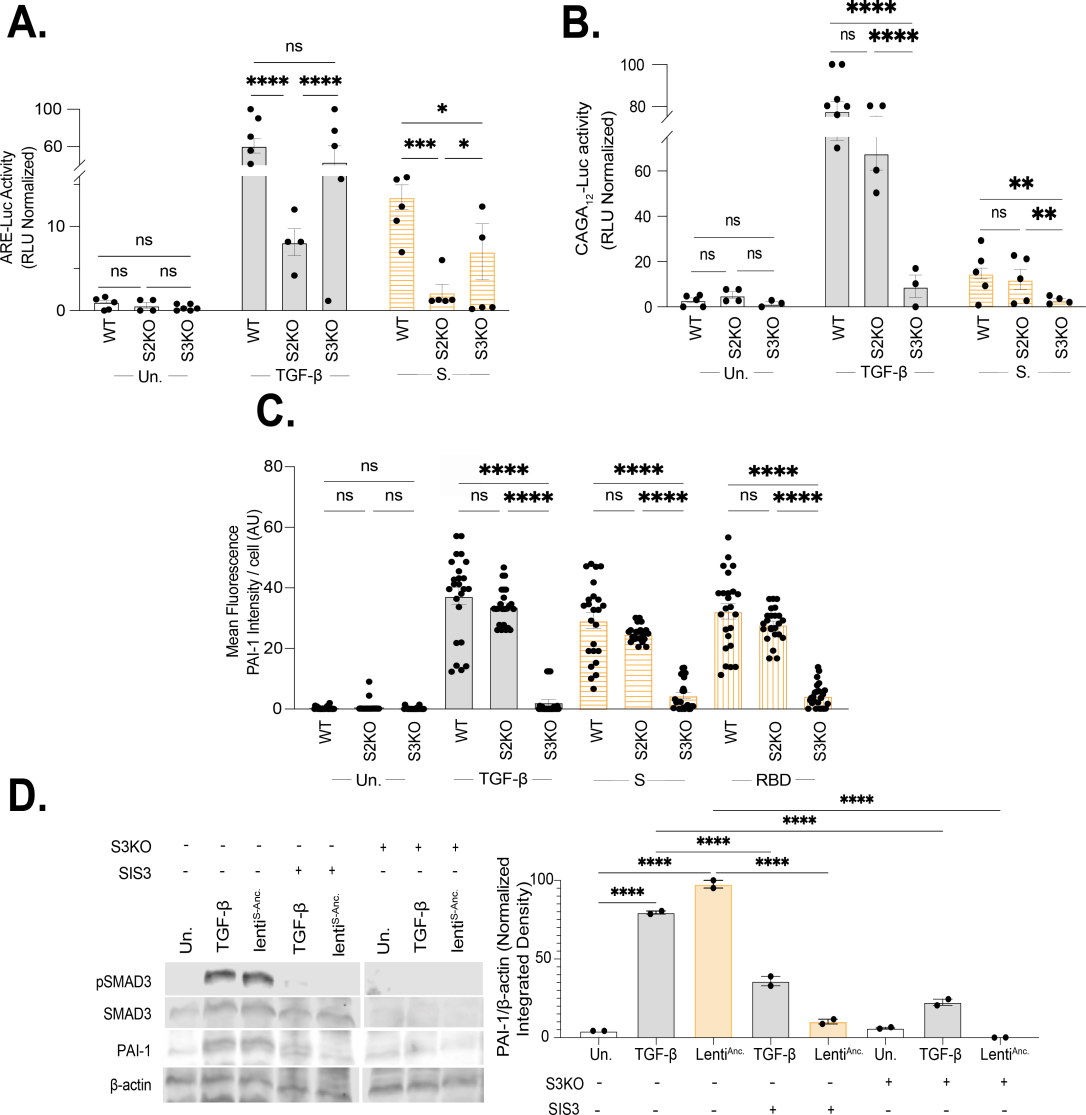
Roles of SMAD2 and SMAD3 in S protein-induced PAI-1 expression. Expression of (**A**) ARE-Luc (SMAD2/4) or (**B**) CAGA_12_-Luc (SMAD3/4) reporters in HaCaT^WT^, HaCaT,^SMAD2KO^ or HaCaT^SMAD3KO^ was measured following treatment with TGF-β (2 ng/mL) or S protein (150 ng/mL) or left untreated for 24 h. Data were normalized relative to positive (TGF-β) and negative (non-transfected, Renilla) controls. (n = 5, ***P < 0.001 by one-way ANOVA with Tukey’s multiple comparison test). (**C**) Quantification of micrographs (Fig. S4F through H) of HaCaT^WT^, HaCaT^SMAD2KO,^ or HaCaT^SMAD3KO^ cells treated with TGF-β (2 ng/mL), S (105 ng/mL), or RBD (200 ng/mL) proteins or left untreated for 24 h. Images are representative of ten technical replicates from two biological replicates. Data were quantified by measuring the mean fluorescence intensity of PAI-1 per nuclei. (*n* = 24, ****P < 0.0001 by two-way ANOVA with Tukey’s multiple comparison test). (**D**) Expression of pSMAD3, SMAD3, PAI-1, and β-actin (endogenous control) in HaCaT^WT^ or HaCaT^SMAD3KO^ cell lysates following 24 h treatment with either TGF-β (2 ng/mL), lenti^S-Anc,^ or left untreated. Additionally, HaCaT^WT^ cells were treated with SIS3 (10 µM). Blots are representative of two biological replicates (normalized band intensity for each immunoblot was calculated using FIJI (ImageJ2), ***P < 0.001, by one-way ANOVA with Tukey’s multiple comparison test; error bars are representative of mean ± SEM).

To define the pathway by which S protein induces PAI-1, we generated loss-of-function cell lines for SMAD2 and SMAD3 through CRISPR/Cas9 genome editing (Fig. 5A and B). These cells were specifically deficient in SMAD2 (HaCaT^SMAD2KO^) and SMAD3 (HaCaT^SMAD3KO^) expression and signaling (Fig. S4A through D). ARE-Luc and CAGA_12_-Luc are specific reporters and binding motifs for SMAD2/4 complexes and SMAD3/4 complexes, respectively (60–62). Loss of SMAD2 and SMAD3 specifically disrupted induction by TGF-β and S protein of the corresponding reporter assay (Fig. 5A through C). Induction of PAI-1 was strongly attenuated in HaCaT^SMAD3KO^ cells exposed to TGF-β, S, or RBD protein, but was unperturbed in HaCaT^SMAD2KO^ cells (Fig. 5D; Fig. S4F through H). These results are supported by SMAD3-specific inhibitor SIS3 blocking both pSMAD3 activity and PAI-1 induction by S protein (Fig. 5D; Fig. S5). Our data are consistent with previous findings of PAI-1 being downstream of SMAD3 following TGF-β stimulation.

### S protein activates TGF-β signaling by an RGD motif

Consistent with the findings of Biering et al., we have shown that S protein is sufficient to activate TGF-β signaling (33). Biering et al. demonstrated that an RGD peptide phenocopied the activity of S protein in triggering endothelial barrier dysfunction and downregulation of sialic acid expression (33). RGD motifs are ligands for a subset of integrin complexes and can initiate subsequent cellular signaling and remodeling (63). Signaling by RGD to integrins is antagonized by the ATN-161 small-molecule inhibitor. S protein contains an RGD motif in the S1 fragment (residues 403–405) and is highly conserved in early SARS-CoV-2 lineages until the Omicron BA.2 variant. Although Biering et al. implicate the S protein RGD motif in barrier dysfunction, the effects of mutating the S protein RGD motif on TGF-β signaling have not been examined.

**Fig 6:**
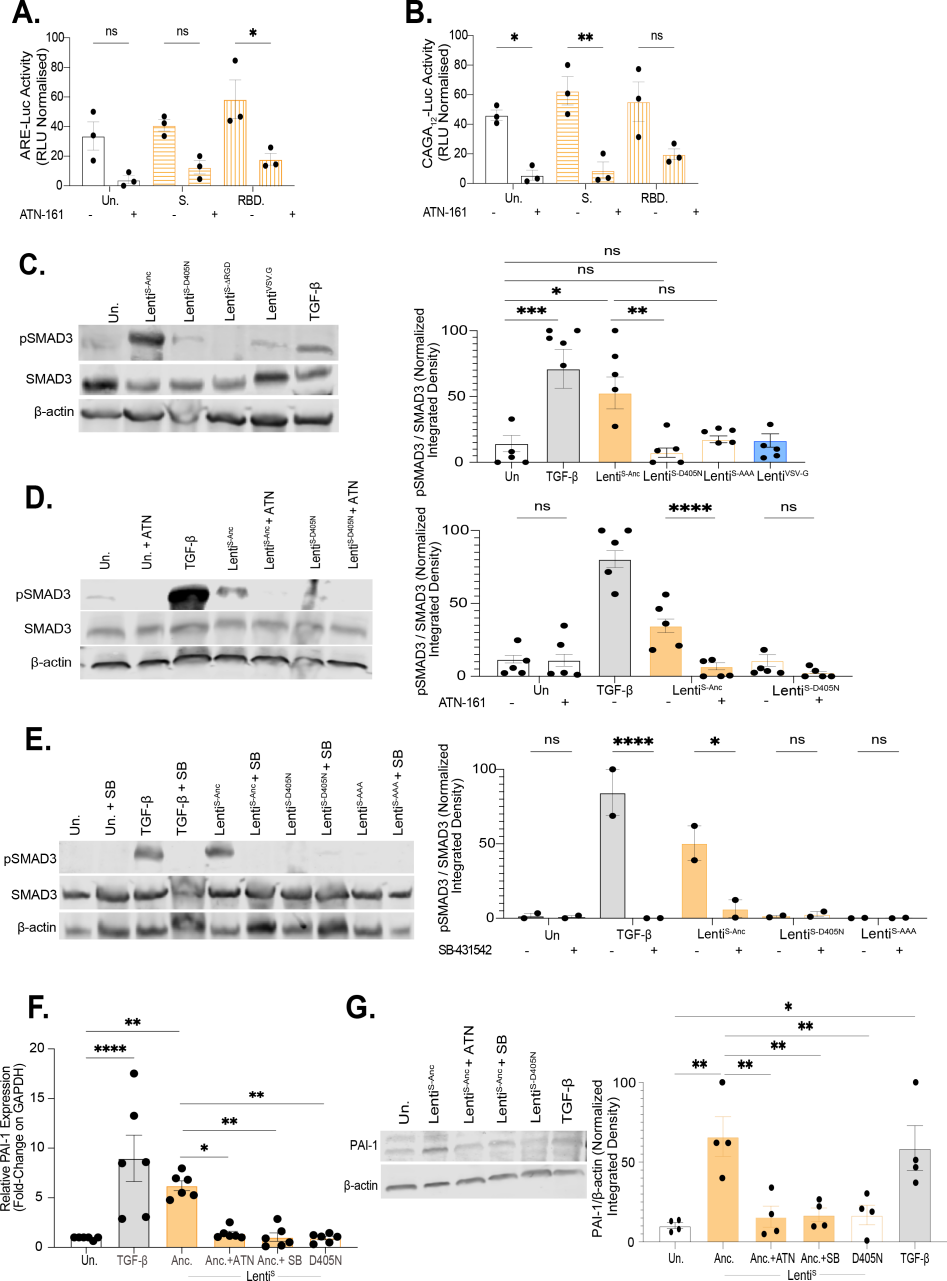
The S protein RGD motif increases the production of TGF-β pathway-dependent PAI-1. (**A**) ARE-Luc (SMAD2/4) and (**B**) CAGA12-Luc (SMAD3/4) reporter constructs were transfected into HaCaT^WT^ cells and treated for 24 h with either TGF-β (2 ng/mL), S protein (105 ng/mL), or RBD (200 ng/mL). Additionally, half of these cells were treated with ATN-161 (20 µM) added 1 h prior to treatment. Reporter activity was normalized to positive (TGF-β) and negative (Renilla-only) controls (n = 3; ***P < 0.001 by two-way ANOVA with Tukey’s multiple comparison test). (**C–E**) HaCaT^WT^ cells were treated for 24 h with TGF-β (2 ng/mL), Lenti^S-Anc^, Lenti^S-D405N^, Lenti^S-AAA^, Lenti^VSV-G^, or left untreated. Western blots show levels of pSMAD3, SMAD3, and β-actin. In panels (**D**) and (**E**), cells were pretreated with ATN-161 (20 µM) or SB-431542 (10 µM), respectively. Densitometry was performed using FIJI and normalized to β-actin. Representative blots from biological replicates are shown (C and D: *n* = 6; E: *n* = 2; ***P < 0.001 by one-way ANOVA with Tukey’s multiple comparisons test). (**F, G**) HaCaT^WT^ cells were serum-starved (0.2% FBS) for 18 h and then treated with ATN-161 (20 µM) or SB-431542 (10 µM) 1 h prior to stimulation with TGF-β (2 ng/mL), Lenti^S-Anc^., Lenti^S-D405N^, or left untreated (MOI = 2). (**F**) RNA was collected at 24 hpi, and PAI-1 (SERPINE1) expression was analyzed by RT-qPCR relative to GAPDH using the ∆Ct method (n = 3; ***P < 0.001 by one-tailed Mann-Whitney test). (**G**) PAI-1 protein levels were assessed by immunoblot at 24 hpi and normalized to β-actin using FIJI (n = 4; ***P < 0.001 by one-way ANOVA with Tukey’s multiple comparisons test).

We observed that ATN-161 potently suppressed induction of SMAD2 and SMAD3 luciferase reporter assays and SMAD3 phosphorylation in response to recombinant S protein and RBD and S protein pseudotyped lentivirus (Fig. 6A, B and D). To determine if the effects of ATN-161 were mediated by the S protein RGD motif and not RGD motifs present in ACE2 and extracellular matrix proteins, we generated an S protein pseudotyped lentivirus carrying the D405N substitution (Lenti^S-D405N^), mutating this motif from RGD to RGN. This mutation was chosen to replicate the residue changes found in the Omicron BA.2 VOC where this RBD site remained structurally functional with a similar binding energy (S-Anc.: −1,780 kcal/mol and S-D405N: −1,794 kcal/mol) but saw a critical acidic residue alteration, which may alter integrin binding capacity (S-Anc.: pH: 6.69 and S-D405N: pH: 7.69) (21). Lenti^S-D405N^ was unable to induce SMAD3 phosphorylation mapping the activity of S protein to this motif (Fig. 6C through E). We generated an S protein pseudotyped virus in which the RGD motif was replaced by three alanine residues (Lenti^S-AAA^), which has been previously shown not to disrupt ACE2 binding or S protein folding (21). Similarly to Lenti^S-D405N^, Lenti^S-AAA^ was unable to induce SMAD3 phosphorylation (Fig. 6D through F). RT-qPCR analysis of SERPINE1 (PAI-1 transcripts) confirmed PAI-1 induction was dependent on an intact RGD motif and was inhibited by ATN-161, SB-431542 (Fig. 6F and G), and SIS3 (Fig. S5E).

### S protein RGD motif inhibits induction of IFN-β via TGF-β signaling

**Fig 7:**
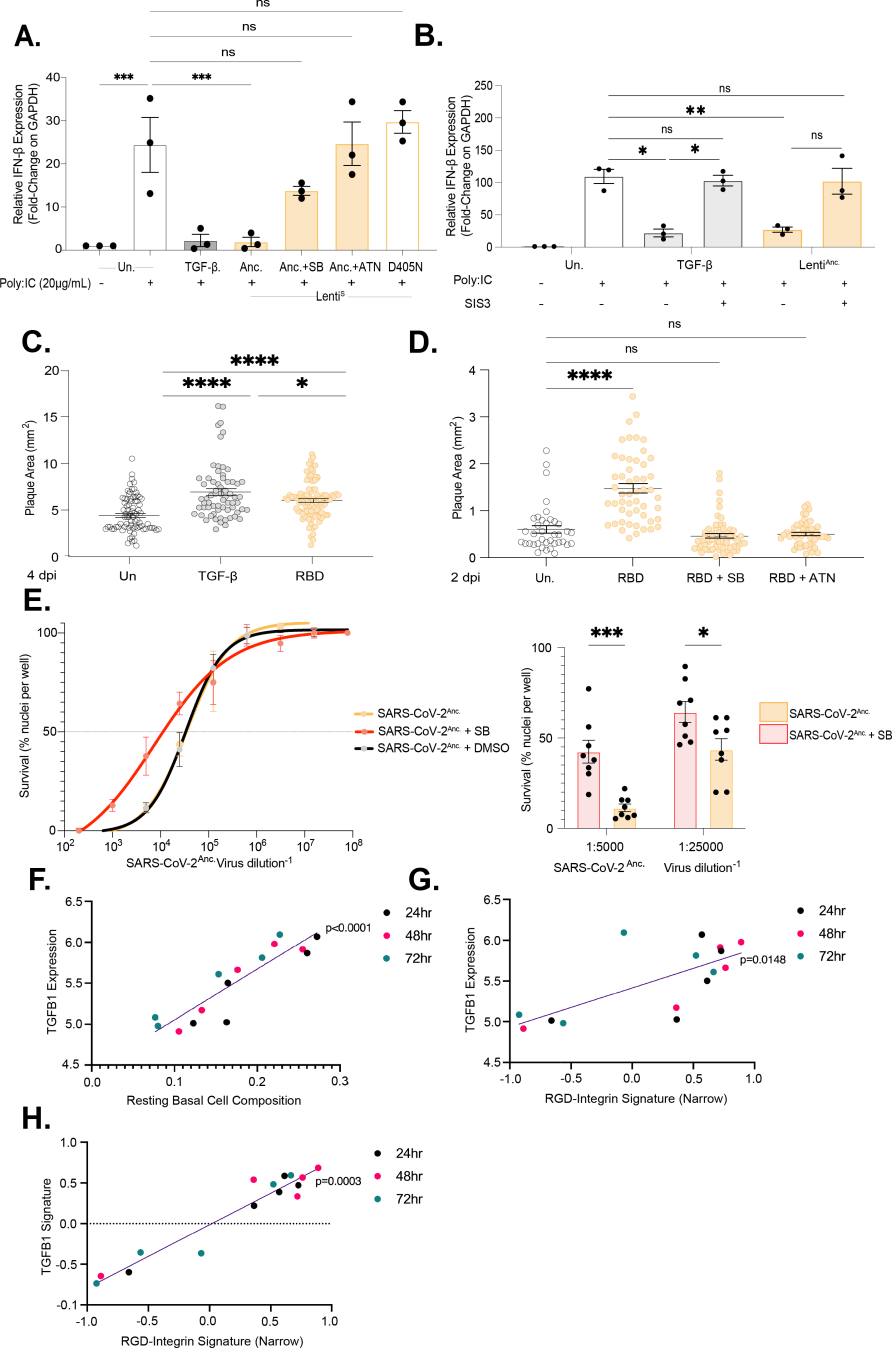
Protein RGD-induced TGF-β signaling suppresses IFN–β and promotes viral spread and cytopathic effect (**A, B**) IFN–β gene expression was assessed in HaCaT cells following treatment with either Lenti^S-Anc^. or TGF-β (2 ng/mL) or left untreated with or without SB-431542 (10 µM), ATN-161 (20 µM), or SIS3 (10 µM). Prior to infection, cells were serum-starved in 0.2% FBS in DMEM for 18 h and then treated with Poly(I:C) (25 µg/mL) for 6 h.. RNA was extracted 24 h post-treatment and analyzed by RT-qPCR relative to GAPDH using the formula ∆∆Ct method. (*n*=3, ***P < 0.001, one-tailed Mann-Whitney test). (**C, D**) A VACV plaque assay was performed on confluent HaCaT cells, which were treated with either TGF-β (2 ng/mL), RBD (10 µg/mL), or left untreated, with or without SB-431542 (10 µM) or ATN-161 (20 µM). After initial treatment, cells were infected with VACV-WR LA-GFP (**C, D**; MOI 0.0003), and the inhibitor treatment was maintained throughout infection. Plaques were measured at either (**C**) 4 dpi or (**D**) 2 dpi. (*n*=4; ***P < 0.001, one-way ANOVA with Tukey’s multiple comparison test). (**E**) Calu-3 cells were treated with SB-431542 (10 µM) or DMSO control and then infected with a serial dilution of SARS-CoV-2 (A.2.2. Strain; 5-fold starting with 1:200). Cell survival was assessed by Hoechst staining and fluorescence microscopy at 72 hpi. Cell nuclei survival was normalized to the uninfected wells (originally seeded at the same density). EC_50_ values (half-maximal cytopathic effect) were calculated by nonlinear regression after fitting a 4-PL sigmoidal curve to each condition (*n* = 8 biological replicates). The 1:5,000 and 1:25,000 dilutions were independently assessed using a two-way ANOVA with Tukey’s multiple comparison test, where *P < 0.05. (F–H) Bulk RNA-seq data from ALI-PBECs from five donors (infected with SARS-CoV-2 at 1 x 10^8^ PFU, 48 hpi; (70) were analyzed for expression of (**F**) basal cell markers (basal cell composition), (**F, G**) TGF-β1, (**H**) TGF-β signature genes, and (**G, H**) RGD-binding integrins (*n* = 5 donors; *P < 0.05, **P < 0.01 by Spearman correlation coefficient (ρ)).

TGF-β is intricately associated with both innate and adaptive immune responses through the regulation of type I interferons by STAT1 and IRF3 signaling cascades (64, 65). To determine if TGF-β signaling induced by the RGD motif exerted an effect on type I interferons, we evaluated IFN-β mRNA expression by RT-qPCR following stimulation with the synthetic dsRNA analog Poly(I:C) (66, 67). Poly(I:C) potently induces IFN-β gene expression, which is suppressed by TGF-β (Fig. 7A). Lenti^S-Anc^ is able to suppress IFN-β induction in a similar manner, and this activity was sensitive to SB-431542, implicating the TGF-β signaling cascade and TβR1 activity. Moreover, sensitivity to ATN-161 and the absence of suppression with lenti^S-D405N^ highlight the importance of the integrin-binding motif RGD (Fig. 7A). We subsequently confirmed this observed IFN-β suppression by SARS-CoV-2 S protein reliant on SMAD3, where in the presence of the SIS3 inhibitor, SMAD3 expression would decrease (Fig. 7B). These results raise the possibility that S protein presenting to cells during SARS-CoV-2 infection might act to suppress local antiviral immunity prior to entry.

### Signaling by S protein RGD motif is proviral

As entry of SARS-CoV-2 downregulates the expression of ACE2, we tested antiviral signaling by the S protein RGD motif with a virus model that does not undergo ACE2-dependent entry (3). We selected Vaccinia virus (Western Reserve strain, WR-VACV) as it has previously been shown to be sensitive to type I interferons *in vivo* and in viral plaque formation (68–70) (Fig. 7C). When treating the cells with TGF-β prior to WR-VACV infection, we observed a significant 1.5-fold increase in the size of the plaques when compared to the untreated control (Fig. 7D, Fig. S6). Treatment with recombinant RBD led to a similar 1.3-fold increase in the size of WR-VACV plaques at either 4 dpi (Fig. 7C) or 2 dpi (Fig. 7D) from the untreated control. If S and RBD protein were acting to promote WR-VACV spread by TGF-β suppression of interferons, we would expect the proviral activity to be sensitive to inhibitors of the TGF-β pathway and RGD antagonists. Cotreatment with either ATN-161 or SB-431542 abolished the effect of RBD, with plaque sizes not significantly different from those of untreated controls at 2 dpi (Fig. 7D). These data are consistent with the S protein RGD motif possessing proviral activity by TGF-β induction suppressing local cellular immunity.

We next examined the requirement for TGF-β signaling in SARS-CoV-2-induced cytopathology in lung epithelial cells. Treatment of Calu-3 cells with SB-431542 rescued cell viability in SARS-CoV-2 (A.2.2)-infected cells (Fig. 7E, *P* < 0.001) and shifted the EC₅₀ by 4.8-fold. Inhibition of SMAD3 using SIS3 trended in a similar direction, increasing EC₅₀ by 4.5-fold (Fig. S7A). Collectively, these data identify TGF-β signaling as proviral during SARS-CoV-2 infection.

The infection of the air-liquid interface human primary bronchial epithelial cell (ALI-HPEC) model offers physiological relevance as it encompasses the differentiation of multiple lung epithelial cell types. By analyzing the previously discussed ALI-HPEC data set by Wang et al., we investigated whether TGF-β signaling is induced in a cell-autonomous manner during infection (71). Bulk RNA-seq data from multiple ALI-PBEC cultures and across three time points (24 h, 48 h, and 72 h) revealed that basal cells, which represent a low proportion of SARS-CoV-2-infected cells (Fig. S7C and D), exhibited strong induction of TGF-β1 expression and a TGF-β signature (72) (Fig. 7F, *P* < 0.0001**,** Fig. S7B). We identified two subsets of integrins: (i) RGD-binding integrins (broad) and (ii) RGD-binding and ATN-161-sensitive integrins (narrow), given our results demonstrating the sensitivity of S protein induction of TGF-β to ATN-161 (Fig. S7B) (21, 22, 73, 74). We also included αvβ1 in the narrow subset despite the lack of experimental evidence for sensitivity to ATN-161 as it has been shown to bind S protein in an RGD-dependent manner (Fig. S7B) (21, 22, 73, 74). In a SARS-CoV-2-infected ALI-PBEC culture, TGF-β1 expression correlated significantly with narrow RGD-binding integrins (Fig. 7G, *P* = 0.01), while a TGF-β1 signature correlated strongly with both broad and narrow RGD-binding integrins (Fig. S7F; *P* =0.001, Fig. 7H, *P* = 0.003).

## DISCUSSION

Here, we describe SARS-CoV-2 S protein-induced TGF-β expression and activation of TGF-β signaling. S protein was delivered to cells through multiple modalities, via a pseudotyped viral model, through treatment with a recombinantly expressed protein or with isolated RBD. All methods induced TGF-β activity and required the presence of ACE2. Prior to our work, induction of TGF-β by S protein in human pulmonary microvascular endothelial cells (HPMECs, lacking ACE2 expression) was described as ACE2-independent (33). We found S protein was able to induce TGF-β at much lower concentrations (100-fold less: 105 ng/mL compared to 10 µg/mL) in an ACE2-dependent manner. ACE2 may act to tether the S protein, allowing for more efficient signaling. Furthermore, both ACE2-dependent and ACE2-independent mechanisms may operate in tandem as the S1 fragment is dispersed during viral entry, allowing different cell populations to respond uniquely based on the S protein concentration and ACE2 expression status.

In response to treatment with S protein and infection with SARS-CoV-2 (Ancestral Strain A.2.2.), we detected TGF-β activity via SMAD2 and SMAD3 reporter assays and SMAD3 phosphorylation, as well as expression of the downstream pro-fibrotic marker PAI-1. By using newly generated SMAD2 and SMAD3 knockout cell lines, we found that PAI-1 induction by TGF-β and S protein required the SMAD3 transcription factor but was independent of SMAD2. Our findings corroborate those of a previous study by Van Tin et al., who observed R-SMAD phosphorylation following S1 treatment (52). We have now mapped this activity to the RGD motif by mutating the S protein RGD motif (lenti^S-D405N^ and lenti^S-AAA^), which abolishes TGF-β activation and signaling. These results align with those of Biering et al., who showed that endothelial barrier dysfunction by S protein could be phenocopied by an RGD peptide or antagonized with ATN-161 (33). Our results could be explained by S protein RGD eliciting the release of latent TGF-β from the latency-associated peptide (LAP) (75–78). This integrin-dependent mechanism regulates homeostasis in the ECM in response to RGD-stimulation of integrin complexes and subsequently releasing active TGF-β from a latent pool (76, 78). There is evidence that TGF-β signaling can drive a positive feedback loop of integrin activation, which may be apparent during SARS-CoV-2 pulmonary inflammation as TGF-β is highly transcribed (28, 74, 79). Intriguingly, α5 and αV integrin subunits, receptors for RGD ligands, are also highly transcribed during SARS-CoV-2 pulmonary inflammation (28, 74). A positive feedback loop would also explain the strong induction of TGF-β observed by RNAseq in response to S protein (33).

In addition to the induction of the pro-fibrotic marker PAI-1, we show that, consistent with the role of TGF-β in immune suppression, S protein signaling through RGD suppressed IFN-β induction. We have provided evidence that S protein exhibits proviral activity with the non-ACE2 binding virus, WR-VACV. Treatment of infected monolayers with S or RBD protein enhanced WR-VACV plaque size, and this effect was abrogated by inhibiting the TGF-β pathway or with an RGD antagonist. The infection for each of the experiments with VACV was done at the same viral titer (Fig. 7C and D; MOI 0.0003); however, we did not see a significant change in the number of infections when treated with S protein in comparison to when treated with TGF-β (Fig. S6C and E). Additionally, a limitation of these results is the use of the VACV model, rather than using authentic SARS-CoV-2 infection. Our assay with VACV has the advantage of isolating S protein activity independently of its role in SARS-CoV-2 entry. However, future research must address the pro-viral activity of the S protein–TGF-β signaling axis through immune suppression during SARS-CoV-2 infection. There is a wealth of data supporting TGF-β inhibition as a possible therapeutic angle for treating SARS-CoV-2, and our findings add another pathway by which TGF-β could act, in addition to endothelial barrier disruption and promotion of fibrosis (33, 41, 42).

Consistent with previous studies, we have shown that the TGF-β receptor inhibitor SB-431542 reduced cytopathology of SARS-CoV-2-infected Calu-3 cells (33, 80). We propose that S protein RGD motif binding to integrins primes cells to viral infection via induction of TGF-β. This is supported by the ALI-PBEC model where a TGF-β transcriptional response is tightly correlated with the expression of RGD-binding integrins. Previous studies have reported elevated TGF-β expression and suppressed type I interferon responses during SARS-CoV-2 infection, consistent with our findings (33, 40, 81–84). Notably, infection ALI-PBEC cultures revealed that SARS-CoV-2, unlike other human coronaviruses, fails to induce robust IFN responses while promoting the expression of inflammatory cytokines (71). TGF-β expression has been shown to inhibit STAT1 signaling and IRF3 phosphorylation, both essential for inducing type I IFNs (83). Furthermore, SMAD3 has been found to impair IRF3 function by suppressing target genes (85). In contrast, SMAD2/3 expression has also been observed to elevate IFN-β in respiratory syncytial virus-infected macrophages, suggesting that the effects of TGF-β may vary depending on the cell type and context (48, 86). Future studies will be required to determine the extent to which TGF-β suppression of type I interferons guides the outcome of SARS-CoV-2 infection.

Our data pinpoint the key role of the RGD motif in S protein-induced cellular signaling. Although RGD motifs are well-characterized ligands for integrin complexes, the availability of the S protein RGD to signal to integrins is contested. Structurally, the RGD motif is located adjacent to the ACE2 interaction interface within the RBD, potentially masking it during virus entry (87, 88). Other studies have suggested that multiple conformational shifts in S protein during RBD presentation, specifically to the “up” and “down” conformation, allow exposure of the RGD without obscuring the ACE2-binding surface (89).

The RGD antagonist, ATN-161, can rescue mice from a lethal dose of SARS-CoV-2, inhibit endothelial barrier dysfunction induced by SARS-CoV-2, and restore viability in infected cells *in vitro* (28, 29, 33). All these studies were performed with the Ancestral Strain of SARS-CoV-2 that carries an intact RGD motif in S protein; it should be noted, however, that ACE2 also harbors an RGD motif. Our results demonstrate that the D405N S protein mutation phenocopies ATN-161 treatment and thereby implicates an S protein–integrin–TGF-β signaling axis.

Functionally, integrin complexes have been implicated with SARS-CoV-2 attachment and entry (21–23, 90). Many viral entry pathways involve the expression of integrin ligands as surface proteins, such as the RGD motif, to engage with membrane-bound integrin receptors (10, 91, 92). Recent studies have shown that RGD-containing SARS-CoV-2 viruses can infect ACE2-negative cells, suggesting that integrins may assist in viral entry (21, 90). Bugatti et al. show that mutating the RGD motif or infecting with Omicron BA.5, a variant lacking the RGD motif, removes this alternative entry route and decreases the expression of αVβ3 integrin complexes (90). This has led to speculation that non-RGD viruses, such as Omicron VOCs (BA.2 onward), may not only lose the ability to bind to these integrin complexes for entry but potentially account for the change in tissue tropism observed in these viruses (90, 93–95). In contrast, the S2 fragment has been shown to upregulate integrin complex α5β1, leading to enhanced ACE2-dependent cell–cell fusion (96). Zhang et al. demonstrated that an S protein with mutated RGD >RGA motif binds at equal affinity to α5β1 integrin complexes when compared to the Ancestral Strain (96). RGD-independent binding implicates interactions with other integrin complexes that may occur during viral entry (10–12, 96, 97).

With almost one in five patients suffering from sequelae associated with long COVID, many studies have already suggested TGF-β signaling, and the RGD motif could be targets for potential therapeutics (98). A recent study found that integrin inhibitor GLPG-0187, an inhibitor of integrin-mediated activation of TGF-β, still functions within the Delta and Omicron variants (87, 99). Additionally, both S protein antibodies and circulating S protein have been reported in COVID-19 patients up to 6 months post-infection, potentially contributing to long COVID sequelae (100).

This study enhances our understanding of the molecular link between SARS-CoV-2 and TGF-β signaling. We have demonstrated that the SARS-CoV-2 S protein RGD motif induces TGF-β expression, leading to SMAD2/3 signaling in an ACE2-dependent manner. Canonical TGF-β signaling upregulated the pro-fibrotic marker PAI-1 expression specifically through SMAD3. Additionally, TGF-β signaling was found to suppress IFN-β induction, contributing to immunosuppression and establishing a proviral state. Understanding the signaling capacity of S protein in the context of viral infection, and in isolation, is a promising strategy for developing novel treatments.

## MATERIALS AND METHODS

### Cell lines

Human keratinocytes (HaCaT^WT^; RRID: CVCL_0038) and Human Embryonic Kidney cells (HEK293T^WT^, ATCC CRL-3216) were maintained in Dulbecco’s modified Eagle’s medium (DMEM; Life Technologies). All media were supplemented with 10% fetal bovine serum (FBS), 292 µm/mL L-glutamine, 100 units/mL penicillin, and 100 unit/mL streptomycin (DMEM-FPSG) and incubated at 37°C and 5% CO_2_. Human epithelial lung adenocarcinoma cells (Calu-3; ATCC HTB-55) were used for authentic SARS-CoV-2 (A.2.2 variant) live viral infection. HEK293T^+ACE2^ were sourced from Stuart Turville.

### CRISPR-Cas9 editing of SMAD2 and SMAD3

gRNAs for SMAD2 and SMAD3 were designed using the CRISPR design tool (101) along with online gRNA design tools, Benchling and eCRISPR, for paired gRNA nickase knockouts. gRNAs were cloned into the nickase mutant pSpCas9n(BB)−2A-GFP vector (PX461, Addgene #48140), and integration of the insert was verified via Sanger sequencing (AGRF, Westmead). HaCaT^WT^ cells were transfected with the SMAD2 and SMAD3 gRNA integrated plasmids and sorted for GFP expression by FACS (BD FACSAria IIu).

#### gRNA sequences

5′ -GGGTGGAGAAGTCTATTGGGAAAG-3′ (SMAD2 Guide 1)

5′ -ACAAGAGGCTGTTTTCCTAGCGT-3′ (SMAD2 Guide 2)

5′ -AGTGGTGATGGCTTTCTCAAGCTC-3′ (SMAD2 Guide 3REV)

5′ -CATCACTTTTTACAGGCAACTTG-3′ (SMAD2 Guide 4REV)

5′-GGCTAGCACTTCGCGGACGA-3′ (SMAD3 Guide 1)

5′-AAGTGAGGGGGCTAGCACTT-3′ (SMAD3 Guide 3Z)

5′-CCCGATCGTGAAGCGCCTGC-3′ (SMAD3 Guide 4Z)

5′-GAAGGGCGAGCAGAACGGGC-3′ (SMAD3 Guide 2)

After FACS enrichment, cells were split into different dilutions; either 2 cells per well or 14 cells per well, and these cell clones were expanded. After suitable expansion from 96-well plates to 6-well plates, DNA and protein samples were extracted from 10 different cell clones. PCR primers were designed using Benchling to verify SMAD2 or SMAD3 from multiple different clones using high-fidelity Q5 polymerase PCR (primers listed below). This experiment was run in parallel to immunoblotting with rabbit anti-SMAD3 (Cell Signaling Technologies) to confirm mixed-cell populations for the absence of SMAD2 or SMAD3.

#### Primer sequences

5′-GGGTGGAGAAGTCTATTGGGAAAG-3′ (SMAD2 P1FOR)

5′-ACAAGAGGCTGTTTTCCTAGCGT-3′ (SMAD2 P2FOR)

5′-AGTGGTGATGGCTTTCTCAAGCTC-3′ (SMAD2 P3REV)

5′-CATCACTTTTTACAGGCAACTTG-3′ (SMAD2 P4REV)

5′-CGGAGGGATCTGCGCATCAAAGC-3′ (SMAD3 P1FOR)

5′-GAAACGCAAAGACACCACCACCT-3′ (SMAD3 P1REV)

5′-AGAAACGCAAAGACACCACCACCTC-3′ (SMAD3 P2REV)

5′-TTGCATGAAACACAGACTGGGA-3′ (SMAD3 P3FOR)

### Pseudotyped lentivirus production

Lenti^S-Anc^ and Lenti^VSV.G^ used in Fig. 2, 4 and 6 were constructed as previously described in (102, 103). Lenti^S-Anc^, Lenti,^S-D405N^ and Lenti^S-AAA^ were constructed separately with the same protocol used in Fig. 6 and 7. HEK293T cells were grown to 75% confluency (1.75 × 10^7^ cells in a T75 flask) prior to transfection. Pseudotyped virus particles were generated by co-transfecting a S protein expression construct (0.36 µg/mL; with either: pCG1-SARS-2 ∆18, pLVX-S.Anc, pLVX-S.D405N, and pLVX-S.AAA) or a VSV.G control plasmid (pHCMV-G), a GFP-luciferase vector (0.72 µg/mL; with either pCDH-GFP or pBCKS-GFP) and with either a single lentivirus packaging plasmid (0.54 µg/mL; ps.PAX2, Fig. 6 and 7) or a three-plasmid tat, gagpol, and rev system (pcDNA.31tat101mL, pHCMVrevmlwhvpre, and pHCMgagpolmlstwhv, Fig. 2, 4 and 6) into HEK293T^WT^ cells, using OptiMEM (Gibco) low serum media supplemented with either Fugene HD (Promega) or Lipofectamine 2000 (Invitrogen, Thermo Fisher Scientific). Following overnight incubation, low serum media was replaced with DMEM supplemented with 10% FBS and 1% PSG. Forty-eight hours post-transfection, the supernatant was removed and centrifuged at 200 x rcf for 5 min to pellet cell debris and additionally passed through a 0.45 µm syringe filter. The viral filtrate was concentrated via the addition of one volume of Lenti-X (Takara Bio USA, Inc) to three volumes of the filtrate and stored at 4°C overnight. The final mixtures were spun down at 3,200 x rcf for 30 min. Viral pellets were resuspended in 1.5 mL SFM.

### Lentivirus titration

HEK293T^+ACE2^ cells were plated at 70% confluency in 96-well plates. A 1:2 dilution series of lentivirus samples was incubated with the cells for 1 hour in a final volume of 100 µL with SFM. Following incubation, the infection media containing the pseudovirus was replaced with DMEM supplemented with 10% FBS and 1% PSG. Cells were maintained in culture for 2 days to allow for complete pseudo-infection, transduction, and expression of the GFP transgene. Wells were imaged on the Nikon Eclipse Ti-E Microscope, and the number of fluorescent cells was segmented via Ilastik (1.4.1b7-arm64) and subsequently selected and quantified Fiji/ImageJ (2.16.0/1.54 p). The fluorescent titer of the pseudotyped lentivirus was estimated by the expression of GFP in particulate infectious units/mL (PFU).


$$viral\;titer\;\left(TU/mL\right)\;=\;\frac{no.\;of\;green\;fluorescent\;particles\;\times dilution\;factor}{volume\;of\;virus\;\left(mL\right)}$$

Additionally, the viral titer was quantified using the Lenti-X p24 Rapid Titre Kit (Takara Bio USA, Inc.; obtained from Scientifix) as per the manufacturer’s instructions. For p24 measurements of lenti^S^ Pseudotyped viruses, viral supernatant samples were diluted 1:100,000 after initially performing a titration. Samples were analyzed using a CLARIOstar Plus Plate Reader (BMG LABTECH) at 450 nm and the CLARIOstar Plus (v6.20, 15.09.2022) Reader software and MARS (v.20, 15.09.2022) data acquisition software. From the absorbance values and the concentrations of p24 determined via the constructed standard curve, the following equation was used to determine the infectious units / mL (IFU/mL) of lentivirus, based on 1 LP containing 8 × 10^−5^ pg of p24.


$$\begin{array}{c} 1\;LP=\frac{2000\times 24\times 10^{3}Da}{6\times 10^{24}}=8\times 10^{-5}pg\text{ of }p24\\ LP/mL=10^{7}\times IFU/mL\times 1000\approx 10^{10}\;LP/mL\\ p24\;(ng/mL)=\frac{LP/mL}{1.25\times 10^{7}}\approx 800\;ng/mL \end{array}$$

### Pseudotyped virus infection

HaCaT^WT^, HEK293T,^+ACE2^ or HEK293T^WT^ cells were seeded at a density of 1.25 × 10^6^ cells per well in a 6-well plate and inoculated with pseudotyped virus at an MOI of 5. Pseudotyped virus infection was performed by “spinoculation” at 800 x rcf at 35°C for 1 h. Cells were left at 37°C with 5% CO_2_ for either 2 days for SDS sample buffer cell lysis for immunoblot or 3 days before fixation in 4% paraformaldehyde for immunofluorescence. TGF-β treatment at 2 ng/mL (Peprotech Pty. Ltd. and Sigma-Aldrich Pty. Ltd.) was included as a positive control for pathway induction. Where treatment with TβR1 inhibitor SB-431542 (10 µM; Sigma-Alrich Pty. Ltd.) was required, cells were pretreated for 1 h.

### RNA extraction, cDNA synthesis, and quantitative PCR

Quantitative PCR was run on HaCaT^WT^ cell culture samples infected with multiple SARS-CoV-2 pseudotyped viruses*.* Cells were washed 24 hpi with PBS to remove remaining serum, cellular debris, and suspended cells. Adherent cells were detached using trypsin and pelleted at 200 x rcf for 5 min. The supernatant was aspirated, and RNA from these cell culture pellets was extracted using the RNA Isolate II mini kit (Meridian Biosciences Inc.) following the manufacturer’s instructions. The resulting yield was estimated by the NanoDrop1000 (Thermo Fisher Scientific, Australia) and ~500 ng of RNA was reverse transcribed into cDNA using iScript SuperMix (Bio-Rad Laboratories Inc.). Quantitative PCR was carried out using the SYBR Select Mastermix (Applied Biosystems) following the primers provided below:

5′-CCGGAACAGCCTGAAGAAGTG-3′ (PAI-1 FW)

5′-GTGTTTCAGCAGGTGGCGC-3′ (PAI-1 REV)

5′-ACAACTTTGGTATCGTGGAAGG-3′ (GAPDH FW)

5′-GCCATCACGCCACAGTTTC-3′ (GAPDH REV)

5′-AAACTCATGAGCAGTCTGCA-3′ (IFN-β FWD)

5′-AGGAGATCTTCAGTTTCGGAGG-3′ (IFN-β REV)

PCR was performed on the QuantStudio6 Pro Instrument (Thermo Fisher Scientific, Australia). Technical replicates were averaged and normalized against GAPDH. Data were obtained from four biological replicates (12 technical replicates) and expressed as fold change ∆∆CT from the respective control groups.

### Plaque assay and WR-VACV infection

WR-VACV LifeAct(LA)-GFP was chosen as an unrelated dsDNA viral model to investigate the impact of SARS-CoV-2 S protein on HaCaT cells. Lifeact is a 17-amino acid peptide that is used to visualize actin, and the GFP tag allows for visualization of the F-actin cytoskeleton in live cells (104). Lifeact-GFP was previously generated and cloned in VACV genes C10L and C11R by homologous recombination, as previously described in (70). Infections with WR-VACV were conducted using a standard methodology. Initially, the growth medium was aspirated from the cell cultures, followed by a single wash with warm PBS. Subsequently, each virus was diluted to the required MOI in DMEM without FBS (serum-free medium, SFM). This diluted virus was then applied to the cells. The cells were incubated with the virus mixture for 1 h at 37°C, 5% CO_2_. Post-incubation, the virus inoculum was removed, and cells were washed once with warm PBS and then overlaid with fresh growth medium consisting of a 1:1 mixture of 2 x MEM (supplemented with 10% FBS and 1% PSG, Gibco) and 1.5% carboxy-methyl cellulose (CMC, Sigma-Alrich Pty. Ltd.). Plaques were imaged at either 2 or 4 dpi under 488 nm light via a ChemiDoc plate imager (BioRad) and quantified using Fiji/ImageJ (2.14.0/1.54 f).

### Protein treatments

Several protein treatments were used as either controls or assessed during this research. Each of the proteins used was maintained in DMSO and frozen for extended storage according to indicated instructions either from the supplier or the manufacturer. For TGF-β treatment, cells were treated similarly to viral infections, where TGF-β (2 ng/mL; Peprotech) was added to both the SFM and recovery medium. Inhibition of TGF-β signaling or SMAD3 phosphorylation during infection was achieved by including SB-431542 (10 µM; Tocris) or SIS3 (10 µM; Selleck) in the infection and recovery medium. For signaling-based experiments, S protein and RBD proteins were treated at 105 ng/mL and 200 ng/mL, respectively, within 10% DMEM, and cells were left for 24 h before transcriptional or protein-based experiments. In plaque assays, S protein and RBD were treated at various concentrations from 0.1 to 20 µg/mL (0.1 µg/mL, 1 µg/mL, 5 µg/mL, 10 µg/mL, and 20 µg/mL) after serum starvation (0.1% FBS) for 24 h.

Recombinantly produced SARS-CoV-2 S protein HexaPro and SARS-CoV-2 receptor binding domain (RBD) proteins were provided by the Drug Discovery Initiative (University of Sydney). The SARS-CoV-2 S protein HexaPro expression plasmid was a gift from Jason McLellan (Addgene plasmid, https://www.addgene.org/154754/). The HexaPro version of the SARS-CoV-2 S protein was selected as it possesses six beneficial proline mutations that confer improved expression and stability of the S protein trimer relative to the parental protein. The gene also contains a C-terminal HRV3C-His_6_-Strep tag to enable the purification of the protein. SARS-CoV-2 S protein HexaPro was expressed in EXPI293F cells via transient transfection of the expression plasmid using polyethyleneimine (PEI, 25 kDa). Cultures were incubated at 37°C in 5% CO_2_ with orbital shaking at 130 rpm for 96 h following transfection. At 96 h post-transfection, cells were harvested via centrifugation at 4,000 × rcf for 20 min, and the supernatant was separated and filtered using a 0.22 µm filter. SARS-CoV-2 S protein HexaPro in the supernatant was purified by immobilized metal affinity chromatography (IMAC) using Ni-NTA agarose. Following IMAC, the protein was dialyzed into a buffer comprising 2 mM Tris pH 8.0 and 200 mM NaCl and was further purified via size-exclusion chromatography (SEC) using a HiLoad 16/600 Superdex 200 column (Cytiva). The protein was eluted from the SEC column using a buffer containing 2 mM Tris, 200 mM NaCl, pH 8.0, concentrated, and stored at −80°C. The protocol used was adapted from references 53, 54.

SARS-CoV-2 RBD (residues 319–541 of SARS-CoV-2 S protein flanked with the N-terminal signal peptide (residues 1–14) from the native SARS-CoV-2 S protein to enable secretion of the expressed protein and a C-terminal His_6_-tag to enable purification) was produced using an expression vector kindly provided by Dr. Florian Krammer (Icahn School of Medicine, Mt Sinai) (105, 106). This plasmid was used to transiently transfect EXPI293F cells using PEI (25 kDa). Cultures were incubated at 37°C in 5% CO_2_ with orbital shaking at 130 rpm for 72 h following transfection. Supernatants were separated via centrifugation at 4,000 × *g* for 20 min 72 h post-transfection. SARS-CoV-2 RBD in the supernatant was purified using Ni-NTA agarose pre-equilibrated in 20 mM NaH_2_PO_4_ pH 8.0, 500 mM NaCl, and 20 mM imidazole. The protein was eluted from the Ni-NTA agarose using a buffer comprising 20 mM NaH_2_PO_4_ pH 7.4, 300 mM NaCl, and 500 mM imidazole. SARS-CoV-2 RBD-containing fractions from IMAC were pooled, concentrated, and purified further via size exclusion chromatography (SEC) using a Superdex 200 10/30 Gl column (Cytiva). SARS-CoV-2 RBD was eluted from SEC column using a buffer containing 20 mM HEPES pH 7.4 and 150 mM NaCl, concentrated, and stored at −80°C.

### Immunofluorescence imaging

HaCaT, HEK293T^+ACE2,^ or HEK293T^WT^ cells were seeded onto round or square glass coverslips; poly-L-lysine solution (0.1% wt/vol, Sigma, Australia) was added to coverslips before HEK293T^+ACE2^ or HEK293T^WT^ cells were seeded. When cells were 80% confluent, infection or treatment was carried out as previously described. Upon reaching the designated timepoint, coverslips were fixed with paraformaldehyde (PFA) in cytoskeletal buffer (CB) [10 mM 2-(N-morpholino) ethanesulfonic acid (MES) buffer, 0.15 M NaCl, 5 mM ethylene glycerol tetraacetic acid (EGTA), 5 mM MgCl2, 50 mM glucose, pH 6.1].

Post-fixation, cells were permeabilized in 0.1% Triton X-100 in CB, followed by 20 min in blocking buffer (1% (vol/vol) bovine serum albumin (BSA), 2% (vol/vol) fetal bovine serum in 1X CB). Cellular protein PAI-1 was visualized using anti-PAI-1 (Santa Cruz Antibodies, C-9) or fluorescently tagged lenti^S-Anc^. Primary antibodies were left for 30 min at 1:200 dilution. The corresponding secondary antibody was added at 1:1,000 dilution for 30 min. Finally, slides were stained in DAPI diluted in CB (1 µg/mL) for 2 min and then mounted on glass slides with Mowiol mounting media (10% (wt/vol) polyvinyl alcohol 4–88, 25% (wt/vol) glycerol).

Slides were visualized using an Olympus BX51 Microscope with Reflection Fluorescence System, Mercury Burner (U-RFL-T), F-view monochrome fluorescence camera and DAPI (347 nm/442 nm [#31013 v2]), FITC (495 nm/515 nm [#31001]), and TxRed (594 nm/610 nm [#31004]) Chroma filters. Micrographs were captured using AnalySIS LS Starter (Olympus Soft Imaging Systems, v2.8) and processed using Fiji/ImageJ (2.14.0/1.54 f).

PAI-1 activity was enumerated by measuring the mean fluorescent intensity per individual cell in three replicate images via ImageJ. Figure corrections for color blindness were completed in ImageJ, where blue was changed to magenta, green was changed to yellow, and red was changed to green. For each of the measurements, regions of interest (ROIs) were chosen based on the cell nuclei, where Alexa Fluor 568 was indicative of the detection of PAI-1. Results were then normalized in comparison to the negative (untreated/uninfected) and TGF-β control.

### Luciferase assay

HaCaT or HEK293T cells were transfected with firefly-luciferase reporter and Renilla control to perform a luciferase assay, as described previously by the literature (107). The firefly-luciferase reporter plasmids used within this study included SMAD2-dependent ARE-Luc, SMAD3-dependent SBR_6_-Luc, and SMAD3/4-dependent CAGA_12_-Luc.

To obtain the highest transfection efficacy in cells and prevent cytotoxicity, the transfection conditions were optimized with 0.5 µg of Luciferase reporter used and 0.005 µg of Renilla control plasmid diluted in SFM. Lipofectamine 2000 (Invitrogen, Thermo Fisher Scientific) was used as a transfection reagent where 3 µL was used for every 1 µg of DNA and diluted in SFM. This reagent was left initially for 5 minutes and then combined with DNA (including reporter assays) for a subsequent 30 min. DNA and Lipofectamine 2000 solution were then applied to cells in SFM and left for 4–6 h at 37°C. Media was subsequently aspirated and replaced with 10% DMEM and left for 24 h allowing for effective transfection to occur.

Luciferase reporter assays were performed in cells transfected with each of the reporter plasmids (107–109). Cells were infected or treated after reaching 80% confluence, and infection/treatment time differed: 8 h (TGF-β) or 24 h (S protein, RBD). After infection/treatment time, cells were lysed using Passive Lysis Buffer (Promega, USA) to extract firefly-luciferase and Renilla in each sample. Using a TD-20/20 luminometer (Turner Designs), the light emission of firefly-luciferase was divided by Renilla to determine the relative amount of luciferase present in each sample.

### Immunoblots

Cells were washed in PBS and harvested on ice in sodium dodecyl sulfate (SDS)-reducing sample buffer (62.5 mM Tris-HCl, 0.25 M glycerol, 2% SDS, 0.01% bromophenol blue, 12.5% β-mercaptoethanol) and boiled three times at 95°C for 3 min. Proteins were separated by SDS-polyacrylamide gel electrophoresis (SDS-PAGE) in running buffer (25 mM Tris Base, 190 mM glycine, 0.1% SDS, pH 8.3) using 15 well-precast mini-PROTEAN gels (BioRad, USA). Separated proteins were transferred to a nitrocellulose membrane (Hybond-C Extra; Amersham Biosciences) by electroblotting using Transfer Buffer (48 mM Tris, 39 mM Glycine, 20% methanol, 0.04% SDS).

Membranes were probed with primary antibodies in TBST-Milk (5% milk in tris-buffered saline, TBS with 0.1% Tween 20) or TBST-BSA (4% BSA in TBS with 0.1% Tween 20) for the detection of proteins. A primary monoclonal antibody specific to SMAD3 (C67H9), phosphorylated SMAD3 (D12E11), or SMAD3-dependent protein PAI-1 (SC-5297) was assessed. Anti-β-actin (Sigma-Aldrich, AC-74) was used as a cellular control. Membranes were washed three times in TBST-Milk or TBST-BSA and then probed with appropriate secondary antibodies conjugated to either IRDye 650LT or IRDye 800CW. These stain-free secondary antibodies were then exposed to UV light for 45 minutes to excite the proteins, providing a fluorescent signal and image from an LI-COR Odyssey CLx image scanner (Millennium Science). Images were captured using ImageLab Software and processed using Fiji/ImageJ (2.14.0/1.54 f).

### Live SARS-CoV-2 infection for immunoblot analysis and cell survival assay

To assess TGF-β signaling induced by the SARS-CoV-2 Ancestral Variant (A.2.2), live virus experiments were conducted under biosafety level 3 (BSL-3) conditions at the Kirby Institute, UNSW, Australia. Live SARS-CoV-2, initially isolated from diagnostic respiratory specimens that tested RT-qPCR positive, was propagated in VeroE6TMPRSS2 cells at an MOI of 0.025 and incubated for 24 h before the supernatant was collected, cleared, and frozen. Calu3 cells were seeded in a six-well plate and grown to 80% confluency. These cells were subsequently infected with SARS-CoV-2 at an MOI of 0.01 and left for 72 h. Prior to infection, half of the cells were treated with 10 µM SB-431542 for 1 h, and after the media was replaced during infection, the inhibitor was maintained at the same concentration. The same lysis procedure was performed as detailed in the immunoblot section using (SDS)-reducing sample buffer. Subsequent immunoblot analysis was conducted in BSL-2 conditions.

To assess the role of TGF-β signaling in SARS-CoV-2 Ancestral Variant (A.2.2)-induced cell death, the same live viral infection reagents and conditions as the immunoblot analysis were utilized. The methodology for these experiments was adapted from prior work by the Turvlle Lab and Anupriya Aggarwal (110, 111). Calu-3 cells were seeded in a 384-well plate and grown to confluency. An hour before infection, eight independent biological cell replicates were treated with either SB-431542 (10 µM), DMSO (0.1% vol/vol), or left untreated. These cells were infected with eight subsequent 5-fold serial dilutions (starting with 1:200) of virus and left for 72 h. Following incubation, Hoechst-33342 live nuclear dye (5% vol/vol; Invitrogen, R37605) was added to the wells 2 h prior to imaging on the InCell Analyzer HS2500 high content microscope (Cytiva). The entire well surface was imaged, and nuclei were enumerated using fluorescence microscopy. Fluorescence images were processed with IN Carta analysis software (Cytiva) to obtain total nuclei counts per well. For survival curves and EC_50_ calculations, nuclei counts were normalized so that 100% represents the average cell number for mock-infected controls and 0% represents the average cell number for the highest viral concentration test, using Min-Max standardization.

A dose-response curve was generated by plotting % survival against viral concentration (fivefold log dilution). A nonlinear regression model using a 4PL sigmoidal curve (dose-response curve with variable slope and four parameters) was implemented to plot curves for each of the three treatment conditions (SB, DMSO, and untreated). Select dilutions were chosen for a direct comparison between SB-treated and untreated conditions to assess statistical significance.

### Isolation, growth, and infection of primary bronchial epithelial cells (PBECs)

Primary human bronchial epithelial cells (PBECs) were isolated as described in the previous study by Wang et al. (71) To examine cell-type specific responses and intercellular signaling during SARS-CoV-2 infection in an air-liquid interface model, we re-analyzed single-cell RNA-seq data from ALI-cultured primary human airway epithelial cells (ALI-PBEC) derived from five adult donors infected with SARS-CoV-2 (A.2.2. Strain, 3 × 10^5^ PFU) and harvested at 48 hpi. Cell populations were annotated based on published markers, focusing on the basal and ciliated cell populations. For each donor, we quantified five variables at the single-cell level: basal cell proportion, TGF-β1 and type I IFN transcript abundance, TGF-β pathway signature, and expression of RGD-binding integrins (described in Fig. S7B). A scatterplot for three independent time points from each of the patients' ALI-PBEC models was constructed for bivariate data and correlation analysis.

The TGF-β signature used was based on a bulk-RNA data set (GSE84135, (72)), in which cultures of submerged airway epithelial cells (basal cells) were treated with TGF-β and measured for gene expression over 72 hours. The DESeq2 differential expression script from this study was adapted, where significantly upregulated genes (BH-adjusted *P*-value < 0.05, log2foldChange > 1) were considered TGF-β-responsive. The full list of genes used in the GVSA and comparisons is listed below:

*LAMC2, ITGB6, FN1, SERPINE2, TGFBI, SERPINE1, ABCA1, VIM, NEAT1, MALAT1, TALAM1, COL4A1, KRT14, LAMA3, PMEPA1, HTRA1, SLITRK6, SKIL, TPM1, TNC, THBS1, COL1A1, SERPINB5, COL7A1, LTBP2, IGFL1, ITGAV, PLAU, FSTL3, COL4A2, STON2, FLRT2, JAG1, NT5E, DIXDC1, PLEK2, FSTL1, HMGA2, ITGA5, SPARC, AMIGO2, SOX4, IVNS1ABP, TMSB4X, PTPRK, F2RL1, CHD9, FERMT1, LIMA1, TGFBR1, NLRP1, ITGB1, SPOCK1, ITGA6, MCAM, ADAM19, GJB2, GPCPD1, FAT1, JUN, VCAN, BMPR2, ATP1B1, NAV1, SNAI2, MFAP2, CALD1, BLTP1, COL17A1, ADAMTS7, TAGLN, ARHGAP21, LAMB3, VMP1, TTN, KCNJ15, DSC2, SULF2, ZFP36L1, CD59, PTPRM, TUT4, KRT16, MBOAT2, STK38L, HMGA2-AS1, TUBB3, JMJD1C, DUSP10, ITGA2, PTHLH, SCD,* and *GOLGB1.*

### ALI-PBEC bulk RNA-seq deconvolution

To infer the relative abundance of respiratory epithelial cells within ALI-PBEC bulk RNA-seq data sets, cellular deconvolution was performed using CIBERSORTx (112). A custom single-cell reference, derived from unpublished ALI culture data sets curated by the Faiz Lab (RBMB, School of Life Sciences, UTS), was used to distinguish resting basal, suprabasal, multiciliated, club, and goblet cell populations. Raw counts were normalized to counts per million (CPM), followed by batch-corrected deconvolution using S-mode with 100 permutations as parameters. The output was returned as proportional estimates (e.g., “0 .XX”) for each cell type per sample.

These proportions were then paired with gene-level expression data from the same RNA-seq samples to generate bivariate data sets, enabling correlation analyses between cell type abundance and transcriptomic features. Spearman correlation coefficient (ρ) was calculated for each donor across the five cell types, with statistical significance assessed for each comparison, where the null hypothesis aligned with rho = 0 or no correlation. Correlation results were then aggregated across the cohort to identify consistent patterns of association.

### ELISA

For indirect ELISA analysis of cell supernatants for detection of TGF-β, HaCaT cells were grown to confluency in six-well dishes prior to 24 h of serum starvation (using SFM). At this point, TGF-β or S protein treatments, or Lenti^S-Anc^. or Lenti^VSV.G^ infections were applied in SFM, as previously described. Supernatants were collected at specified time points and used alongside the Human TGF-β1 ELISA kit (Sigma-Aldrich) according to the manufacturer’s instructions. A standard curve was generated using the supplied control standard. Samples were analyzed using CLARIOstar Plus Plate Reader (BMG LABTECH) and the CLARIOstar Plus (v6.20, 15.09.2022) Reader software and MARS (v.20, 15.09.2022) data acquisition software.

### Statistical analysis

Data were collected and plotted in GraphPad Prism 10 (Version 10.5.0, GraphPad Software, LLC). Error bars represent the mean ± the Standard Error of the Mean (SEM) unless reported otherwise. The significance comparison between groups differed for each set of data. Plaque assays used unpaired *t*-test, IFA relative fluorescence analysis, and normalized luciferase activity using a one-way ANOVA and Tukey’s multiple comparison test. Values of *P* < 0.05 were considered statistically significant.

The normalization of luciferase activity values was performed based on the cell line, and the data were taken from and relative to the positive (TGF-β treated) and negative (uninfected, non-treated, non-transfected) controls for each sample as transfection efficiency varied. Min-Max standardization was used to satisfy the requirements to provide the data with values between 0 and 1.

For RT-qPCR experiments, each biological replicate represents the average of six technical replicates, performed at both the standard and ⅛ dilution concentrations. C_t_ values were first normalized to the GAPDH housekeeping gene (∆C_t_), and fold changes were calculated using the ∆∆C_t_ method relative to the uninfected control.


$$\Delta \Delta C_{t}=\Delta C_{t}\;\left(target\;sample\right)-\Delta C_{t\;}\left(control\;sample\right)$$

From this value, the fold change in comparison to the uninfected samples (which was denoted as 1) was calculated. The significance was assessed using a one-tailed Mann-Whitney test (a nonparametric test).

Sigmoidal dose-response curves and EC_50_ values were obtained with GraphPad Prism 10 (Version 10.5.0, GraphPad Software, LLC) using the nonlinear regression for inhibitor versus response with variable slope and four parameters.

For bulk RNAseq data, each correlation was assessed using Spearman correlation coefficient (rho) for each donor across the five variables. Statistical significance was determined for each correlation, and results were aggregated across the cohort to identify consistent patterns.

For all tests, *P < 0.05, **P < 0.01, ***P < 0.001, and ****P < 0.0001.

## Data Availability

All data generated during this current study are included in the article or within the supplemental material.

## SUPPLEMENTAL MATERIAL

Fig. S1 jvi.00435-25-s0001.docx Immunoblotting S protein induced SMAD3 activity over time.

Fig. S2 jvi.00435-25-s0002.docx S protein and RBD activate SMAD3 and PAI-1 in an ACE2-dependent manner.

Fig. S3 jvi.00435-25-s0003.docx Lenti^S-Anc^ activates PAI-1 in cells dependent on the TGF-beta pathway and the presence of ACE2.

Fig. S4 jvi.00435-25-s0004.docx Generation and validation of SMAD2 and SMAD3 knockout HaCaT cells to analyze the roles of each R-SMAD in S protein PAI-1 expression.

Fig. S5 jvi.00435-25-s0005.docx SIS3 inhibits S protein-induced SMAD3-dependent signaling.

Fig. S6 jvi.00435-25-s0006.docx Dose-dependent enhancement of VACV spread by soluble RBD and S protein.

Fig. S7 jvi.00435-25-s0007.docx SARS-CoV-2 live infection in Calu-3 and ALI-PBEC model indicates that TGF-β signaling is active and strongly correlated to RGD-binding integrins.

ASM does not own the copyrights to Supplemental Material that may be linked to, or accessed through, an article. The authors have granted ASM a non-exclusive, world-wide license to publish the Supplemental Material files. Please contact the corresponding author directly for reuse.
