# Supplementary material for: An RGD motif on SARS-CoV-2 Spike induces TGF-β signaling and downregulates interferon
Source: J Virol. 2025 Sep 4;99(9):e00435-25. doi: 10.1128/jvi.00435-25 (PMC12456147; doi:10.1128/jvi.00435-25)
Supplement: Fig. S1 — Immunoblotting S protein induced SMAD3 activity over time. [file jvi.00435-25-s0001.docx]

**
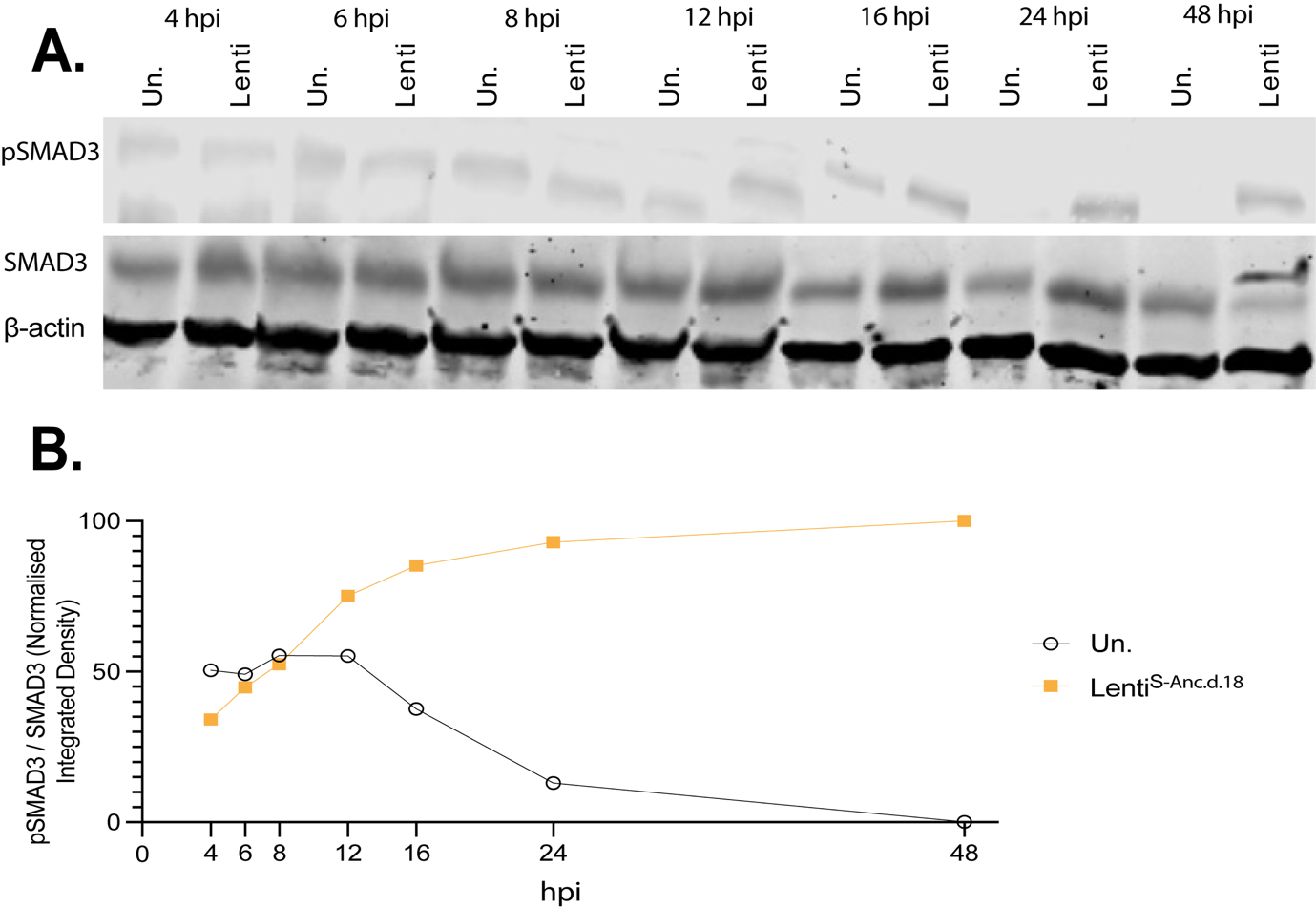
Fig. S1. Immunoblotting S protein induced SMAD3 activity over time**

(A) Expression of pSMAD3 and SMAD3 in HaCaT^WT^ cell lysates following treatment with either lenti^S-Anc^ or left untreated. Lysates were taken after various timepoints including 4 hpi, 6 hpi, 8 hpi, 12 hpi, 16 hpi, 24 Blots are representative of two biological replicates. The Normalized band intensity for each immunoblot was calculated using FIJI (ImageJ2). (n=2, ***p < 0.001 by One-way ANOVA with Tukey’s multiple comparison test). (B) Averages of the normalized band intensity were taken and placed on a line graph to monitor the change of pSMAD3 expression (n=2).
