# Supplementary material for: An RGD motif on SARS-CoV-2 Spike induces TGF-β signaling and downregulates interferon
Source: J Virol. 2025 Sep 4;99(9):e00435-25. doi: 10.1128/jvi.00435-25 (PMC12456147; doi:10.1128/jvi.00435-25)
Supplement: Fig. S2 — S protein and RBD activate SMAD3 and PAI-1 in an ACE2-dependent manner. [file jvi.00435-25-s0002.docx]

#### **
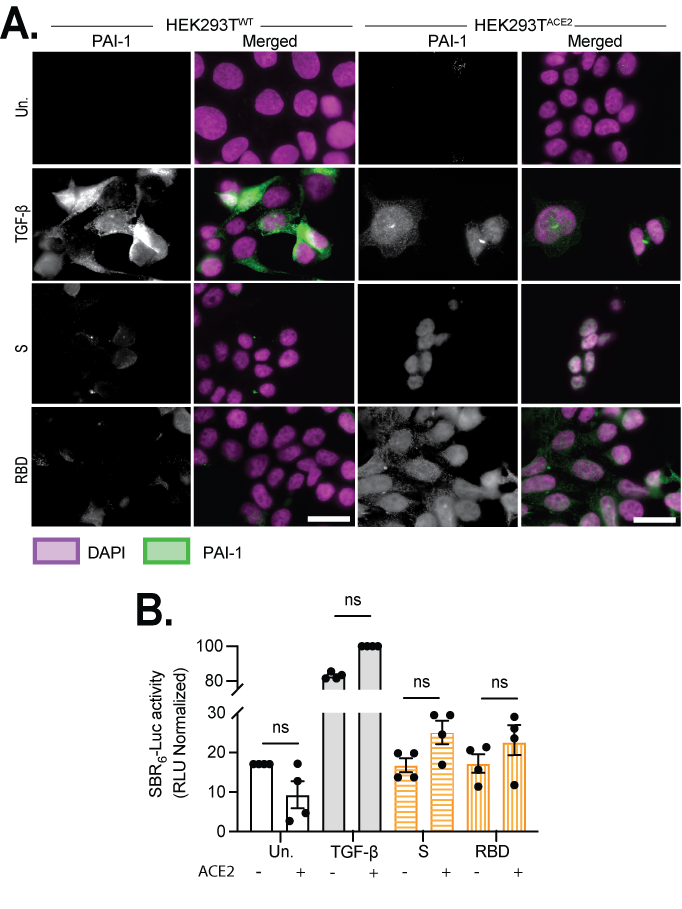
**

#### **Fig. S2. S protein and RBD activate SMAD3 and PAI-1 in an ACE2-dependent manner**

**(A)** Immunofluorescence micrographs of HEK293T^WT^ and HEK293T^ACE2^ cells treated with TGF-β (2 ng/mL), S protein (105 ng/mL) or RBD (200 ng/mL) or left untreated for 24 h. Images are representative of ten fields of view from three biological replicates. SMAD3-dependent protein PAI-1 and nucleic acid (DAPI) are represented in green and magenta, respectively. Scale bar = 50 µm. Data were quantified by measuring the mean fluorescence intensity of PAI-1 per nuclei. **(B)** SMAD3/4 (SBR_6_-Luc) reporter was assayed at 24h in S protein and RBD-treated cells that express (HEK293T^ACE2^) or do not express (HEK293T) ACE2 (**(C)** n = 4, p > 0.05 **(B)** by One-way ANOVA with Tukey’s multiple comparison test).
