# Supplementary material for: An RGD motif on SARS-CoV-2 Spike induces TGF-β signaling and downregulates interferon
Source: J Virol. 2025 Sep 4;99(9):e00435-25. doi: 10.1128/jvi.00435-25 (PMC12456147; doi:10.1128/jvi.00435-25)
Supplement: Fig. S3 — LentiS-Anc activates PAI-1 in cells dependent on the TGF-beta pathway and the presence of ACE2. [file jvi.00435-25-s0003.docx]

#### **
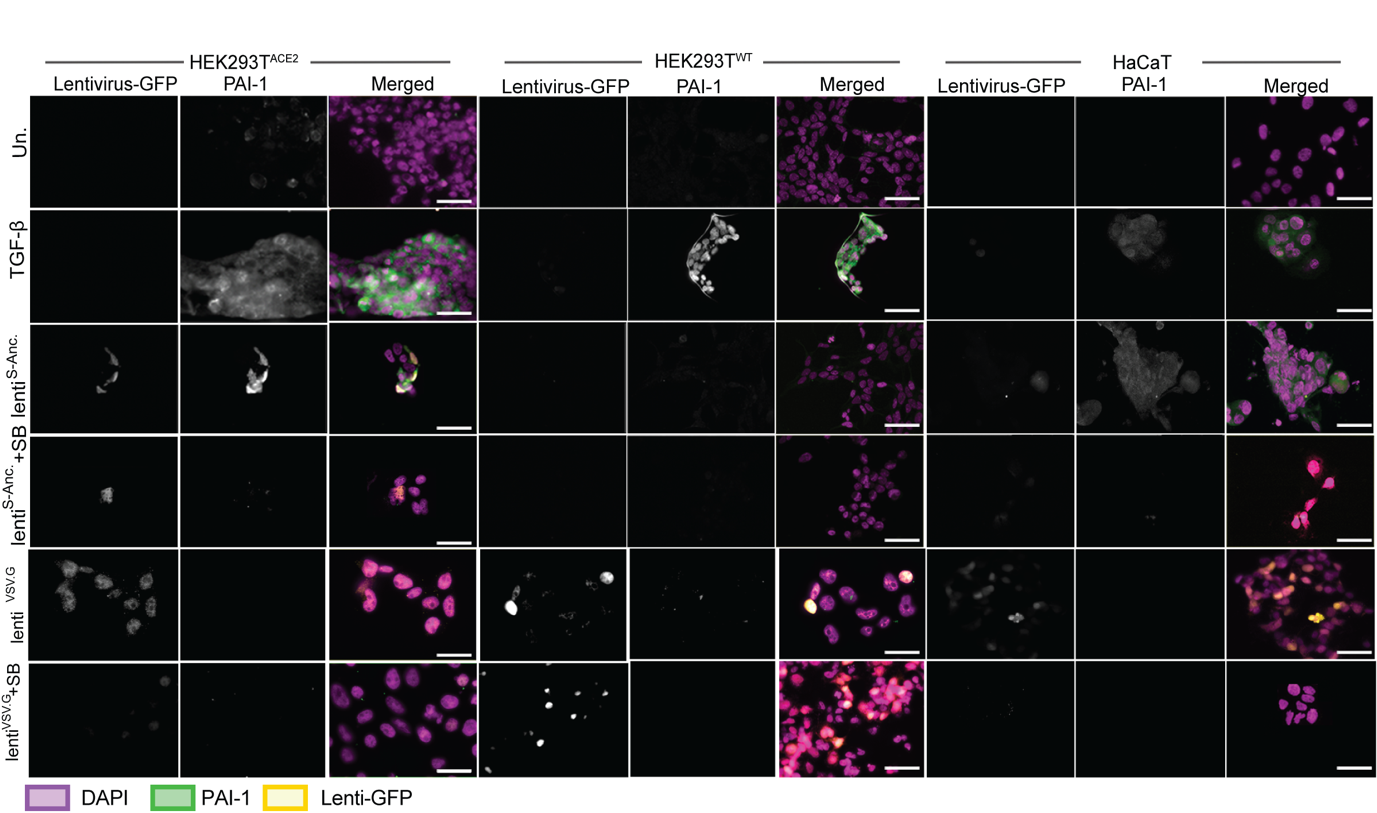
Fig. S3. lenti^S-Anc.^ activates PAI-1 in cells dependent on the TGF-β pathway and the presence of ACE2.**

Micrographs of HaCaT^WT^, HEK293T^ACE2^ and HEK293T^WT^ cells treated with TGF-β (2 ng/mL), infected with lenti^S-Anc.^ or lenti^VSV.G^ or left untreated for 24 h. Additionally, half of the treated cells were supplemented with SB-431542 (10 µM). Images are representative of six technical replicates from three biological replicates. SMAD3/4 dependent protein PAI-1 and nucleic acid (DAPI) are represented in green and magenta, respectively. Lentiviral GFP expression, activated by the model glycoprotein, either being S protein or VSV.G Protein, is represented in yellow. Scale bar = 50 µm. Data were quantified by measuring the mean fluorescence intensity of PAI-1 per nuclei.
