# Supplementary material for: An RGD motif on SARS-CoV-2 Spike induces TGF-β signaling and downregulates interferon
Source: J Virol. 2025 Sep 4;99(9):e00435-25. doi: 10.1128/jvi.00435-25 (PMC12456147; doi:10.1128/jvi.00435-25)
Supplement: Fig. S4 — Generation and validation of SMAD2 and SMAD3 knockout HaCaT cells to analyze the roles of each R-SMAD in S protein PAI-1 expression. [file jvi.00435-25-s0004.docx]

**
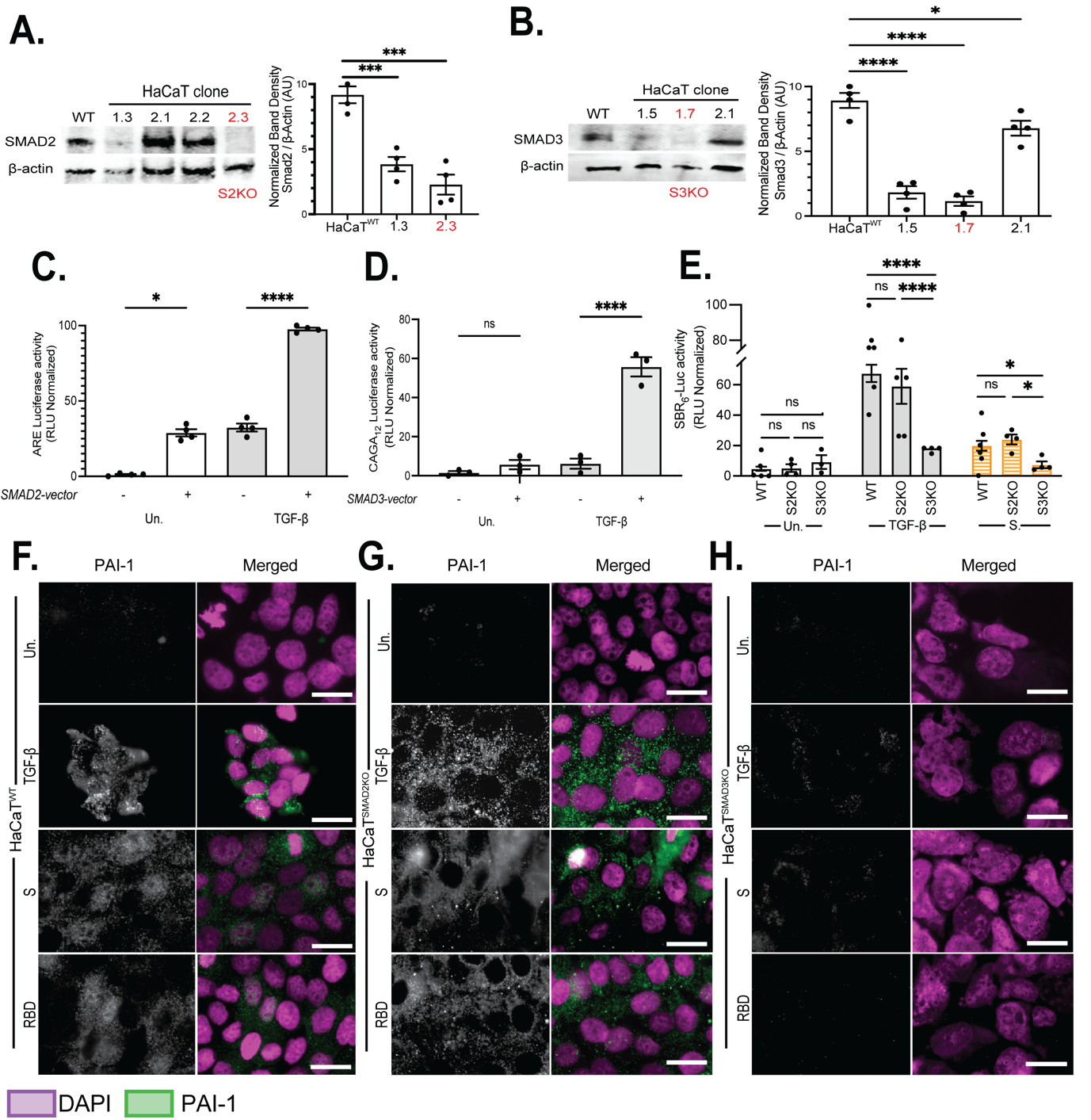
**

**Fig. S4. Generation and validation of SMAD2 and SMAD3 knockout HaCaT cells to analyse the roles of each R-SMAD in S protein PAI-1 expression.**

**(A-B)** Immunoblots confirming loss of SMAD2 (clone 2.3) and SMAD3 (clone 1.7) expression in CRISPR-edited HaCaT lines (n=4 protein lysates for each CRISPR HaCaT clone analysed, ***p<0.001 by One-way ANOVA with Tukey’s multiple comparison test). **(C-D)** ARE-luc (SMAD2/4) and CAGA_12_-Luc (SMAD3/4) reporter activity in knockout lines, with or without rescue by transfected SMAD2 or SMAD3. Cells were treated with TGF-β or left unstimulated. Luciferase values were normalized to Renilla and Min-Mx standardised against TGF-β and unstimulated controls (SMAD2KO: n=4; SMAD3KO: n=4; pairwise t-tests with Bonferroni correction). Expression of **(E)** SBR-Luc (SMAD3/4) reporter activity in HaCaT^WT^, HaCaT^SMAD2KO^ or HaCaT^SMAD3KO^ cells after treatment with TGF-β (2 ng/mL), S protein (105 ng/mL), or no treatment. Data were normalized relative to positive (TGF-β) and negative (non-transfected, Renilla) controls (n=5, ***p<0.001 by One-way ANOVA with Tukey’s multiple comparison test). **(F-H)** Immunofluorescence micrographs of HaCaT^WT^ cells treated with TGF-β (2 ng/mL), S protein (105 ng/mL) or left untreated for 24 h were created from two biological replicates. SMAD3/4 dependent protein PAI-1 and nucleic acid (DAPI) are represented in green and magenta, respectively. Scale bar = 50 µm.
