# Supplementary material for: An RGD motif on SARS-CoV-2 Spike induces TGF-β signaling and downregulates interferon
Source: J Virol. 2025 Sep 4;99(9):e00435-25. doi: 10.1128/jvi.00435-25 (PMC12456147; doi:10.1128/jvi.00435-25)
Supplement: Fig. S5 — SIS3 inhibits S protein-induced SMAD3-dependent signaling. [file jvi.00435-25-s0005.docx]

**
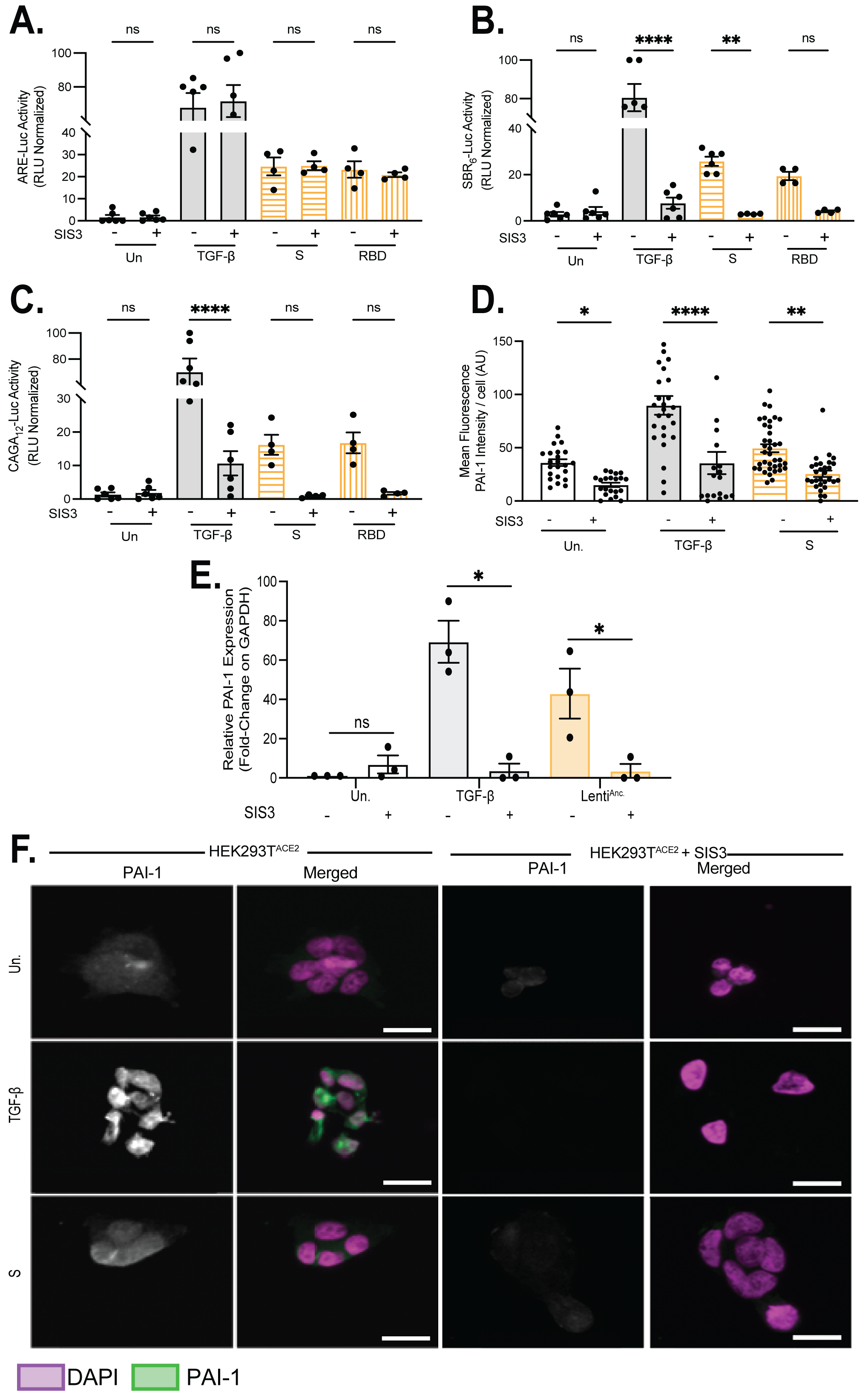
**

**Fig. S5. SIS3 inhibits S protein-induced SMAD3-dependent signalling.**

(**A-C**) Luciferase reporter activity in HEK293T^ACE2^ cells transfected with ARE-Luc (SMAD2/4), SBR_6_-Luc (SMAD3), or CAGA_12_-Luc (SMAD3/4) reporters. Cells were treated with TGF-β (2 ng/mL), S protein (105 ng/mL), RBD (200 ng/mL), or left untreated for 24 h, with or without SMAD3-specific inhibitor, SIS3 (10 µM). Data were normalised to TGF-β (positive) and Renilla-only (negative) controls. (n=6; ****p<0.0001 by one-way ANOVA with Tukey’s test). (**D**) Quantification of mean PAI-1 fluorescence per nuclei in HEK293T^ACE2^ cells treated with TGF-β (2 ng/mL), S protein (105 ng/mL), RBD (200 ng/mL), or left untreated for 24 h, with or without SMAD3-specific inhibitor, SIS3 (10 µM). (n=12; ***p<0.001 by two-way ANOVA with Tukey’s multiple comparison test). (**F**) Representative immunofluorescence images of treated HEK293T^ACE2^ cells. PAI-1 (green) and DAPI (magenta); scale bar = 50µm. (**E**) PAI-1 mRNA expression following treatment with TGF-β (2 ng/mL), lenti^S-Anc.^, or untreated, with or without SIS3. Cells were serum-starved (0.2% FBS, 18h), pre-treated with SIS3 (10 µm, 1h), and harvested 24 h later for RT-qPCR. Expression was normalised to GAPDH using the ∆∆C_t_ method (n=3; ***p<0.001 by Mann-Whitney test).
