# Supplementary material for: An RGD motif on SARS-CoV-2 Spike induces TGF-β signaling and downregulates interferon
Source: J Virol. 2025 Sep 4;99(9):e00435-25. doi: 10.1128/jvi.00435-25 (PMC12456147; doi:10.1128/jvi.00435-25)
Supplement: Fig. S6 — Dose-dependent enhancement of VACV spread by soluble RBD and S protein. [file jvi.00435-25-s0006.docx]

**
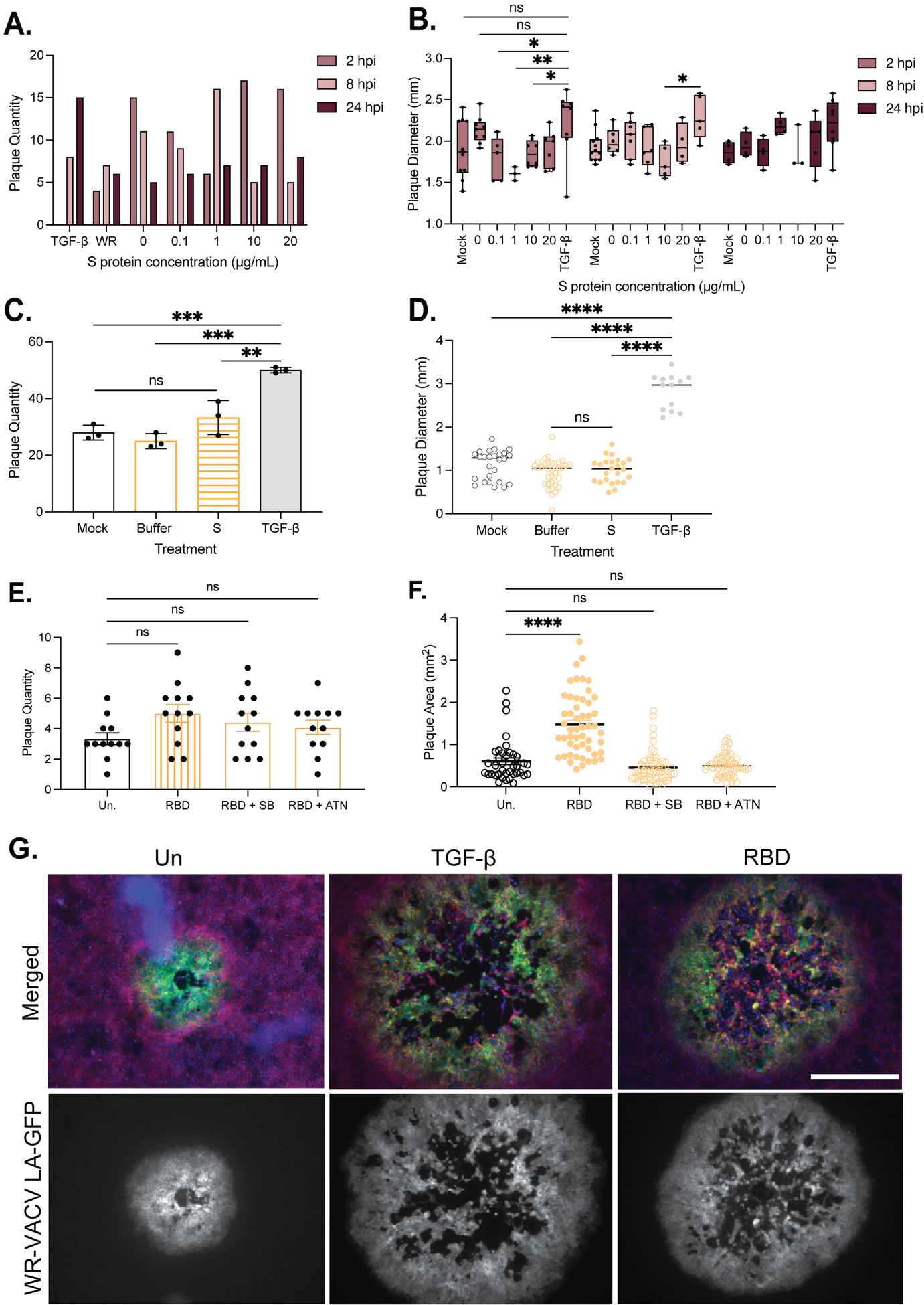
Fig. S6. Dose-dependent enhancement of VACV spread by soluble RBD and S protein.**

(**A-B**) VACV-WR plaque formation in HaCaT cells pre-treated with increasing concentration of S protein or additionally, TGF-β (2 ng/mL) or left untreated. Cells were serum-starved (0.2% FBS) for 24h prior to LifeAct-GFP VACV-WR infection. Plaques were quantified at 5 dpi by FIJI (ImageJ2) (**(A)**: plaque number, n=1; **(B)** plaque diameter, n=3-11; **p<0.01 by one-way ANOVA with Tukey’s test). **(C-D)** Using the results from the concentration gradient, a VACV-WR plaque assay was performed on HaCaT cells treated with either RBD (10 µg/mL), TGF-β (2 ng/mL) (or left untreated) 1 h prior to infection. **(C)** Plaque area and **(D)** Plaque number were assessed at 4 dpi (n=4; ***p<0.001 by one-way ANOVA with Tukey’s multiple comparisons). **(E-F)** RBD-induced enhancement of VACV-WR infection was blocked by co-treatment with SB-431542 (10 µM) or ATN-161 (20 µM). **(E)** Plaque number and **(F)** Plaque area were assessed at 2 dpi (n=2; ***p<0.001; by one-way ANOVA with Tukey’s multiple comparisons). **(G)** Representative Immunofluorescence images of LifeAct-GFP VACV-WR plaques at 48hpi in untreated, TGF-β- (2 ng/mL), RBD-treated (10 µg/mL) HaCaT cells. viral plaques in HaCaT cells after different plaque treatments. GFP (green), DAPI (blue), and Phalloidin-A568 (red). Scale bar = 400 µm.
