# Supplementary material for: An RGD motif on SARS-CoV-2 Spike induces TGF-β signaling and downregulates interferon
Source: J Virol. 2025 Sep 4;99(9):e00435-25. doi: 10.1128/jvi.00435-25 (PMC12456147; doi:10.1128/jvi.00435-25)
Supplement: Fig. S7 — SARS-CoV-2 live infection in Calu-3 and ALI-PBEC model indicates that TGF-β signaling is active and strongly correlated to RGD-binding integrins. [file jvi.00435-25-s0007.docx]

**
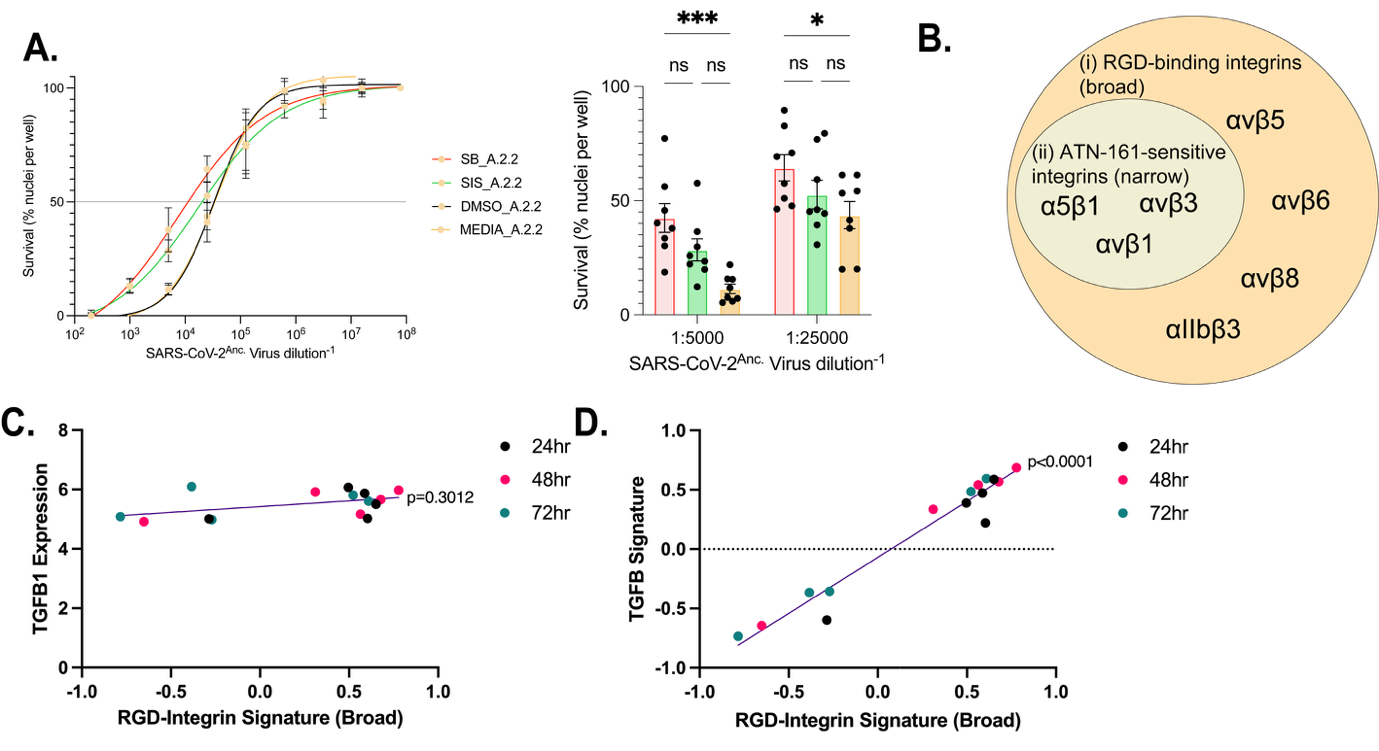
Fig. S7. SARS-CoV-2 live infection in Calu-3 and ALI-PBEC model indicate that TGF-β signalling is active and strongly correlated to RGD-binding integrins**

**(A)** Calu-3 cells were treated with SB-431542 (10 µM), SIS3 (5 µM) or DMSO control, then infected with a serial dilution of SARS-CoV-2 (A.2.2. Strain; 5-fold starting with 1:200). Cell survival was assessed by Hoechst staining and fluorescence microscopy at 72 hpi. Cell nuclei survival was normalized to the uninfected wells (originally seeded at the same density). EC_50_ values (half-maximal cytopathic effect) were calculated by non-linear regression after fitting a 4-PL sigmoidal curve to each condition (n= 8 biological replicates) [(75, 76)](https://www.zotero.org/google-docs/?Uio0y7). The 1:5000 and 1:25000 dilutions were independently assessed using a Two-way ANOVA with Tukey’s multiple comparison test, where *p<0.05. **(B)** A schematic showing the collection of integrin complexes which are defined as either (i) RGD-binding integrins (broad), and (ii) ATN-161 sensitive and RGD-binding integrins (narrow). **(C-D)** Bulk RNA-seq data from ALI-PBECs from five donors (infected with SARS-CoV-2 at 1 x 10^8^ PFU, 48 hpi; [(70)](https://www.zotero.org/google-docs/?XEbEJo)) were analyzed for expression of TGF-β1, TGF-β signature genes and “broad” RGD-binding integrins (n=5 donors; *p<0.05, **p<0.01 by Spearman correlation coefficient (ρ)).
